# Chronic low-grade inflammation drives skeletal aging and neurocognitive decline: inflammaging as a central hub coupling bone–brain aging

**DOI:** 10.3389/fimmu.2026.1874465

**Published:** 2026-09-04

**Authors:** Man Yu, Yu Wu, Ao Wang, Mei Wang, Yao Chen, Hang Ren, Yixin Zhang, Yingjie Liu, Shudong Wang, Xu Fan

**Affiliations:** 1 Health Preservation and Rehabilitation College, Liaoning University of Traditional Chinese Medicine, Shenyang, China; 2 Clinical Laboratory Science College, Liaoning University of Traditional Chinese Medicine, Shenyang, China; 3 Basic Medicine College, Liaoning University of Traditional Chinese Medicine, Shenyang, China

**Keywords:** blood-brain barrier, bone-brain axis, cGAS-STING, cognitive impairment, gut-bone-brain axis, immunosenescence, inflammaging, microglial heterogeneity

## Abstract

**Background:**

Global population aging has driven a marked rise in the co-prevalence of osteoporosis and cognitive impairment, and a bidirectional epidemiological association between the two conditions is now supported by multiple meta-analyses. The shared biological mechanisms underlying this comorbidity, however, remain incompletely defined, and the two disorders continue to be managed within largely separate clinical disciplines.

**Aim and scope:**

This review consolidates existing evidence into an integrative immunopathological framework in which inflammaging—the chronic, low-grade, sterile systemic inflammatory state driven by senescent cells and their senescence-associated secretory phenotype (SASP)—is examined as a shared upstream driver that concurrently reconfigures skeletal remodeling and central neuroimmune dynamics through the bone–brain axis. We do not claim to identify novel molecular targets; rather, we synthesize an integrative perspective that has been treated in disciplinary silos.

**Key mechanistic themes:**

At the molecular level, persistently elevated SASP-derived cytokines (IL-6, IL-1β, TNF-α) engage RANKL-dependent osteoclastogenesis in bone and prime microglial neuroinflammation in the central nervous system. Chronic NF-κB signaling and NLRP3 inflammasome activation, amplified in preclinical models by mitochondrial DNA release via the cGAS-STING axis, sustain this dual pathological output. Within the bone–brain axis, bone-derived endocrine signaling is remodeled during aging: osteocalcin (OCN) secretion declines, while osteocyte-derived sclerostin (SOST) rises and may antagonize Wnt/β-catenin signaling in both compartments. Blood–brain barrier disruption and peripheral immune-cell infiltration further amplify central neuroinflammation.

**Balanced appraisal:**

We explicitly distinguish (i) conceptual hypotheses, (ii) preclinical (cellular and rodent) findings, and (iii) validated human data. Several widely cited mechanisms—including OCN–GPR158-mediated neuroprotection, cGAS-STING-driven neuroinflammation, and microbiome-based longevity signatures—rest predominantly on murine models or single cohorts and require independent human validation. Microglial responses in the aging brain reflect a heterogeneous state space rather than a uniform pro-inflammatory conversion.

**Therapeutic implications:**

Candidate bone–brain dual-targeting interventions—senolytics (dasatinib plus quercetin), NLRP3 inhibitors, cGAS-STING blockade, GLP-1 receptor agonists, and microbiota-targeted strategies—are discussed with explicit reference to current evidence level, safety concerns, and translational limitations, rather than as established co-therapies. Dual-endpoint randomized trials enriched for elevated inflammaging biomarkers are needed before any of these agents can be positioned for clinical use in bone–brain comorbidity.

**Conclusion:**

The inflammaging-centered framework advanced here provides a testable integrative pathophysiological perspective on bone–brain aging comorbidity and a rationale for interdisciplinary “bone–brain integrated” clinical evaluation in older adults, which we frame as an aspirational, hypothesis-generating model rather than an evidence-based standard of care.

## 1 Introduction

### 1.1 The dual burden of osteoporosis and cognitive impairment in an aging world

Global population aging is accelerating at an unprecedented pace. According to the United Nations *World Population Prospects 2024*, the global population currently stands at approximately 8.2 billion and is projected to peak at around 10.3 billion in the mid-2080s. The proportion of individuals aged 65 years and older currently represents approximately 10% of the global population, and this cohort is projected to reach approximately 2.2 billion by the late 2070s, at which point it will surpass the total number of children under the age of 18 (1). Against this demographic backdrop, osteoporosis and cognitive impairment have emerged as two of the most pressing public health challenges in geriatric medicine.

Osteoporosis is the most prevalent metabolic bone disease, characterized by reduced bone mass, deterioration of bone microarchitecture, and a substantially elevated risk of fragility fractures. A systematic review and meta-analysis based on the World Health Organization diagnostic criteria, encompassing 108 studies and 343,704 participants, reported a pooled global prevalence of osteoporosis of 19.7% (95% CI: 18.0%–21.4%) and of low bone mass (osteopenia) of 40.4% (95% CI: 36.9%–43.8%), with both prevalences increasing markedly with advancing age (2). Data from the Global Burden of Disease Study 2019 (GBD 2019) further revealed that disability-adjusted life years (DALYs) attributable to low bone mineral density and related fragility fractures increased by 93.82% globally between 1990 and 2019 (3). The burden of cognitive impairment is comparably formidable: an estimated 57.4 million individuals were living with dementia worldwide in 2019, with a projected rise to approximately 152.8 million by 2050 (4).

#### 1.1.1 Epidemiological convergence

Accumulating evidence indicates that osteoporosis and cognitive impairment are bidirectionally associated rather than co-occurring by chance. A meta-analysis of 136,222 participants reported a significantly elevated risk of cognitive impairment among individuals with osteoporosis (OR = 2.01, 95% CI 1.63–2.48) (5). A reciprocal meta-analysis confirmed that cognitive impairment is independently associated with an increased risk of osteoporosis (RR = 1.56, 95% CI 1.30–1.87), with individuals with Alzheimer’s disease (AD) demonstrating approximately 70% higher odds of osteoporosis than cognitively healthy controls (6). The Rotterdam Study identified low femoral-neck and total-body BMD as independently associated with incident dementia (7), and a population-based cohort of 176,150 community-dwelling older adults confirmed osteoporosis as an independent risk factor for incident dementia (8). **We note, however,** that most of these studies rely on cross-sectional or short-follow-up designs, use heterogeneous outcome definitions (BMD versus clinical osteoporosis; mild cognitive impairment versus all-cause dementia versus AD), and inconsistently adjust for shared confounders (physical activity, vitamin D, sex-hormone status). The causal directionality of the association therefore remains unresolved and is revisited in Section 7.2.

Despite this epidemiological convergence, osteoporosis and cognitive impairment have historically been investigated as independent disease entities, assigned to separate clinical disciplines—namely orthopedics/endocrinology and neurology/geriatric medicine, respectively—with insufficient attention paid to their common mechanistic underpinnings. Prior research has predominantly focused on disease-specific pathways: in osteoporosis, the emphasis has been on estrogen deficiency and OPG/RANKL axis dysregulation; in cognitive impairment, on amyloid-β (Aβ) deposition and tau hyperphosphorylation. The systemic inflammatory mechanisms driving the parallel progression of both conditions have received comparatively little attention. This disciplinary divide perpetuates a dual clinical blind spot: osteoporosis patients rarely undergo cognitive assessment, while systematic bone health evaluation is largely absent in the management of individuals with cognitive impairment.

### 1.2 Inflammaging as a candidate shared upstream driver

The concept of “inflammaging”, a state of chronic, low-grade, sterile systemic inflammation that develops with advancing age, was first introduced by Franceschi and colleagues in 2000 (9), and has since been formally incorporated as a canonical hallmark of aging (10). This state is characterized by chronically elevated circulating levels of pro-inflammatory mediators, including interleukin-6 (IL-6), tumor necrosis factor-α (TNF-α), interleukin-1β (IL-1β), and C-reactive protein (CRP), and is now recognized as a common inflammatory substrate underlying multiple age-related diseases (11). Its principal mechanistic drivers include continuous secretion of pro-inflammatory cytokines by senescent cells through the senescence-associated secretory phenotype (SASP) (12), age-related persistent activation of the NF-κB signaling pathway, and immunosenescence.

In bone, inflammaging has been reported to accelerate age-related bone loss by upregulating RANKL, promoting osteoclastogenesis, and concurrently suppressing osteogenic differentiation of bone-marrow mesenchymal stem cells (13). senescent osteocytes are a major source of local SASP factors (14). These findings, however, derive largely from murine and *in vitro* studies, and their quantitative contribution to human skeletal aging has not yet been formally partitioned. In the central nervous system, chronic peripheral inflammation activates microglia through blood-brain barrier disruption, vagal afferent signaling, and trans-endothelial cytokine passage, and has been implicated in impaired synaptic plasticity, Aβ oligomerization, and tau hyperphosphorylation (15). Of particular mechanistic relevance, the bone-derived hormone osteocalcin (OCN) has been reported in rodent models to cross the blood-brain barrier and engage GPR158 on hippocampal CA3 neurons to enhance spatial memory (16). The translational relevance of this axis in humans is unresolved: independent groups have reported that Bglap/Bglap2-deficient mice on distinct genetic backgrounds do not reproduce the reported cognitive and metabolic phenotypes (17, 18), and human associations between circulating OCN and cognition are inconsistent across cohorts (19). We revisit this controversy in Sections 5.2.1 and 7.2.

### 1.3 Positioning of the present review

Several recent reviews have separately addressed inflammaging in aging biology (11), skeletal osteoimmunology (13, 20), neuroinflammation in cognitive decline (21), and, most recently, the bone–brain axis as an endocrine communication network (16, 19). Our review does not aim to re-describe these fields. Rather, its intended conceptual contribution is threefold:

- to reframe inflammaging as the shared upstream immunological hub that concurrently rewires bone-derived endocrine output (reduced OCN; elevated SOST and FGF23) and central neuroimmune activation, rather than treating skeletal and neurocognitive aging as parallel processes;
- to consolidate the cGAS-STING → NF-κB/NLRP3 → SASP axis as a candidate unifying molecular circuit operating in both tissues, while explicitly noting that human validation of this axis remains at an early stage (22); and
- to translate this integrative perspective into a testable clinical framework for dual bone–brain risk stratification and intervention, framed as hypothesis-generating rather than practice-changing.

### 1.4 Evidence hierarchy adopted in this review

Throughout this review, we distinguish three tiers of evidence: (i) conceptual hypotheses and integrative frameworks; (ii) preclinical findings derived from cellular systems and animal models—principally rodent studies—whose translational relevance requires further validation; and (iii) human data supported by cross-sectional, longitudinal, or interventional studies. Where mechanistic claims are supported predominantly by preclinical evidence, we state this explicitly; inferences to human pathology are framed as tentative. Where widely cited mechanisms are contested (e.g., OCN–GPR158 neuroprotection, cGAS-STING as a driver versus a marker of neuroinflammation, microbiome longevity signatures), the controversy is presented rather than glossed over.

We further acknowledge that the framework proposed here is subject to important limitations, including the near-absence of Mendelian randomization studies with adequately powered instruments, species differences in immunosenescence trajectory that constrain mouse-to-human translation, and the paucity of validated intermediate biomarkers suitable for proof-of-concept intervention trials. These constraints are discussed in a dedicated Section 7.2.

### 1.5 Structure of the review

#### 1.5.1 Literature search and selection strategy

This narrative review synthesizes evidence retrieved through a structured, non-systematic search of PubMed/MEDLINE, Web of Science, and Scopus, complemented by hand-searching of key reference lists. The primary search window was January 2000 to November 2025, with foundational earlier references retained where they defined core concepts, including the original introduction of inflammaging. Search terms were combined across three domains using Boolean operators: (i) inflammaging OR “chronic low-grade inflammation” OR SASP OR “senescence-associated secretory phenotype” OR immunosenescence; (ii) osteoporosis OR “bone loss” OR “bone remodel*” OR osteoclast* OR osteocyte* OR “bone–brain axis”; and (iii) “cognitive decline” OR dementia OR “Alzheimer disease” OR neuroinflammation OR microglia OR “neurovascular unit”. We prioritized mechanistic studies in human tissue or well-characterized rodent models, meta-analyses and prospective cohorts for epidemiological claims, and registered clinical trials or early-phase human interventional studies for therapeutic statements. Editorials, single case reports, and non-peer-reviewed preprints were generally excluded unless the primary literature was sparse. Where multiple reviews covered a subtopic, we cited the most recent authoritative synthesis and added original research where it materially changed the position taken here. This strategy is intended to improve transparency rather than to claim systematic-review exhaustiveness; residual selection bias inherent to narrative synthesis is acknowledged in Section 7.2.1.

The remainder of the review is organized as follows. Section 2 summarizes inflammaging as a functional foundation, focusing on the four molecular nodes (SASP cytokine networks, chronic NF-kB tone, NLRP3 activation, and the mitochondria-cGAS-STING axis) that are re-engaged in later sections. Section 3 examines inflammaging-mediated skeletal aging. Section 4 addresses inflammaging-mediated cognitive impairment, with balanced attention to microglial heterogeneity. Section 5 develops the bidirectional bone–brain axis and identifies inflammaging as its central amplifier. Section 6 consolidates convergent pathological mechanisms (mitochondrial dysfunction, oxidative stress, the gut–bone–brain axis, and insulin resistance). Section 7 summarizes limitations, unresolved mechanistic questions, translational challenges, shared confounding, and clinical implications, and outlines what would be required to convert the proposed framework from a hypothesis into an evidence-based standard of care.

## 2 Inflammaging: a functional foundation for the bone–brain framework

**Figure 1:**
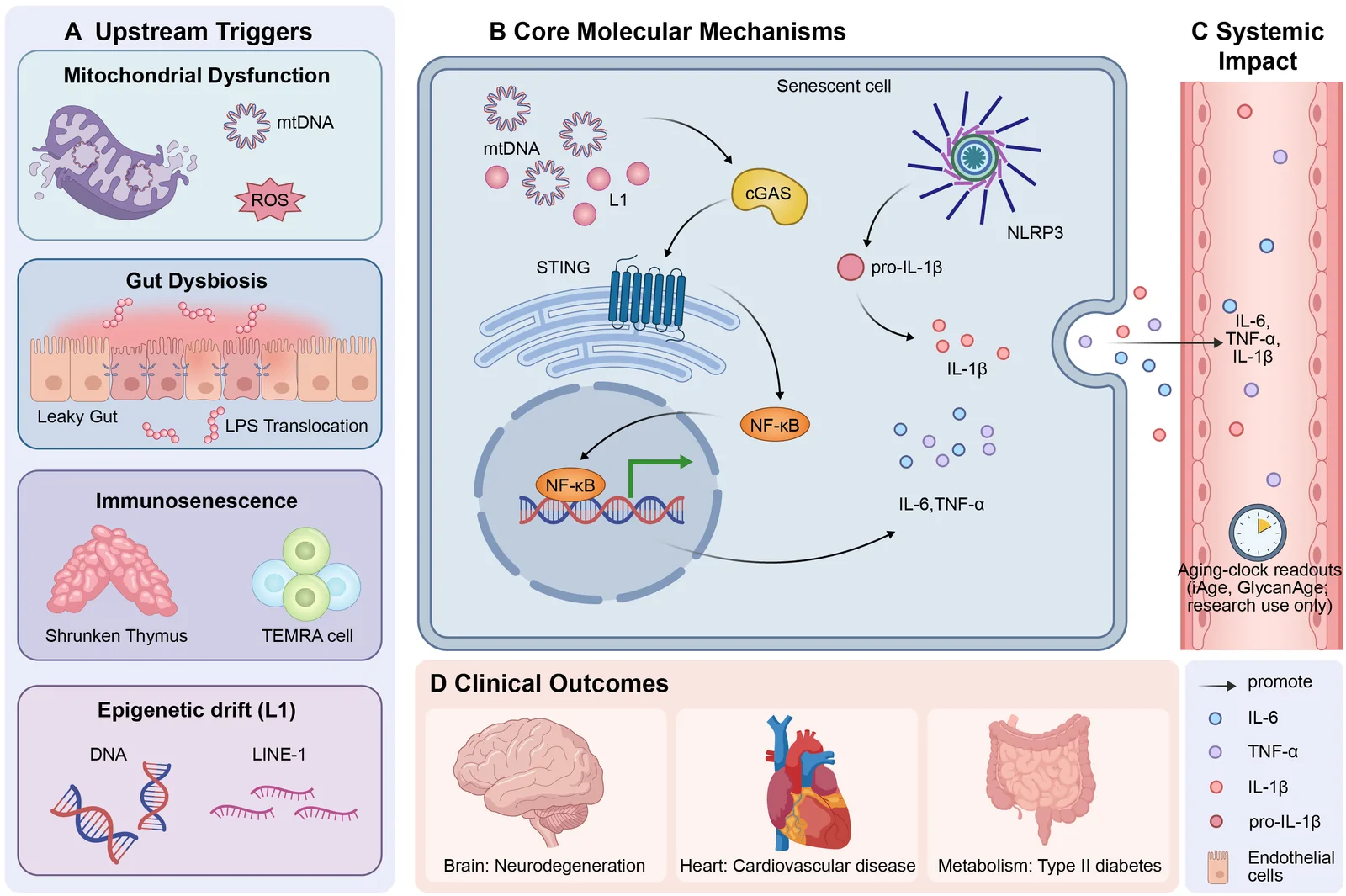
The integrative landscape of inflammaging, from upstream triggers to systemic pathological outputs. **(A)** Upstream triggers include mitochondrial dysfunction, gut dysbiosis with LPS translocation, immunosenescence, and epigenetic drift involving LINE-1 derepression. **(B)** Core molecular mechanisms converge in senescent cells, where mitochondrial DNA release and L1 activity engage cGAS-STING, NF-κB, and NLRP3 inflammasome signaling to promote IL-1β maturation and SASP cytokine release. **(C)** Systemic impact is represented by endothelial-cell exposure to IL-6, TNF-α, and IL-1β and by research-use aging-clock readouts including iAge and GlycanAge. **(D)** Clinical outcomes include neurodegeneration, cardiovascular disease, and type 2 diabetes, presented as downstream multimorbidity associations rather than disease-specific causal proof.

Inflammaging denotes a chronic, low-grade, sterile systemic inflammatory state that accumulates with advancing age and is now recognized as a canonical hallmark of aging (10). It is defined by three cardinal features—chronicity, low intensity, and sterility—and is driven by endogenous damage-associated molecular patterns (DAMPs), senescent-cell secretory products, and metabolic stress signals rather than microbial infection (23). Circulating markers of inflammaging, including IL-6, TNF-α, and CRP, are independently associated with cardiovascular disease, type 2 diabetes, Alzheimer’s disease, sarcopenia, and malignancy (10, 24), and the inflammatory-age index (iAge) has been proposed to capture inter-individual heterogeneity (21). Rather than re-describing the foundational biology of inflammaging in detail—for which comprehensive syntheses are available (11)—this section focuses on the four molecular nodes that are re-engaged in Sections 3–5 as concurrent drivers of skeletal and neurocognitive aging: (2.1) SASP-derived cytokine networks; (2.2) chronic NF-κB tone; (2.3) NLRP3 inflammasome activation; and (2.4) the mitochondria–cGAS-STING axis. Perpetuating factors—mitochondrial dysfunction, gut dysbiosis, immunosenescence, and epigenetic remodeling—are summarized in (2.5) contextualized in their bone- and brain-specific consequences later in the review which is summarized in **Figure 1**.

### 2.1 SASP-derived pro-inflammatory cytokine networks

Chronic elevation of pro-inflammatory cytokines is the most direct molecular hallmark of inflammaging. In aged individuals, IL-1β, IL-6, TNF-α, IL-17, and IL-18 are persistently elevated in both the circulation and tissue microenvironments (25). For the purposes of this review, we highlight IL-6 and IL-1β because both are re-engaged in the bone-remodelling (Section 3.2.1) and neuroinflammatory (Section 4.1) circuits. IL-6 signals through the JAK1/TYK2–STAT3 axis to induce acute-phase protein synthesis, promote skeletal muscle proteolysis, and impair insulin signaling by suppressing IRS-1 tyrosine phosphorylation, tightly coupling inflammation with metabolic dysfunction (26). IL-1β maturation depends on NLRP3 inflammasome activation (Section 2.3); chronic age-related IL-1β elevation contributes to osteoarthritis, neurodegenerative disease, and type 2 diabetes (27).

### 2.2 Chronic NF-κB tone in senescent cells

The NF-κB signaling pathway functions as the master transcriptional regulator of inflammaging (28). Its canonical activation—IκB kinase (IKK) phosphorylation of IκBα, proteasomal IκBα degradation, and nuclear translocation of NF-κB dimers to drive pro-inflammatory transcription—is well characterized and is not re-described here. What is functionally relevant to bone–brain aging is the chronic low-grade activation (“elevated basal tone”) sustained in senescent cells by DNA-damage–driven ATM–NEMO signaling, excessive mitochondrial ROS, and cytosolic mitochondrial DNA sensing via the cGAS-STING axis (Section 2.4) (29). This chronic NF-κB tone will be re-invoked in Section 3.2.1 (as an upstream regulator of RANKL/OPG imbalance) and Section 4.1.1 (as a driver of microglial priming); we therefore do not repeat its molecular anatomy in those sections.

### 2.3 NLRP3 inflammasome activation

The NLRP3 inflammasome assembles from the sensor NLRP3, adaptor ASC, and effector Caspase-1, and requires a canonical two-signal activation model: a priming signal (TLR-driven NF-κB upregulation of NLRP3 and pro-IL-1β) followed by an activation signal (mitochondrial ROS, K^+^ efflux, or particulate DAMPs). Active Caspase-1 cleaves pro-IL-1β and pro-IL-18 and, via gasdermin D pores, drives cytokine release and, in some contexts, pyroptosis (30).

During aging, NLRP3 is maintained in a chronically primed state by mitochondrial ROS, elevated K^+^ efflux, and progressive accumulation of urate and cholesterol crystals in joints and vascular walls; post-translational modifications further lower the activation threshold (31). Chronic NLRP3 activation has been implicated in neurodegenerative disease, osteoarthritis, and type 2 diabetes (32), and selective inhibitors (e.g., MCC950) have shown activity in preclinical models. The translational status of NLRP3 inhibitors is discussed critically in Section 7.3 (**Table 1****);** we note here only that first-generation MCC950 development was constrained by hepatotoxicity signals, and that next-generation compounds remain in early clinical evaluation (46).

**Table 1:** Candidate bone–brain dual-targeting interventions: evidence level and translational limitations.

Intervention	Mechanistic rationale	Current evidence by endpoint	Principal safety/translational concerns
D+Q senolytic combination (dasatinib + quercetin)	SASP clearance	Bone: Phase 2 RCT in postmenopausal women, with BMD and P1NP signal confined to high senescent-cell-burden subgroup (33). Brain: no dedicated cognitive-endpoint RCT; early AD pilot feasibility only (34).	Dasatinib off-target toxicity (cytopenias, pleural effusion, cardiac risk), variable quercetin pharmacokinetics, and unknown long-term safety in older adults.
MCC950 and next-generation NLRP3 inhibitors	Direct blockade of IL-1beta maturation	Bone: preclinical only (35). Brain: preclinical AD models only (36, 37).	Original MCC950 development constrained by hepatotoxicity signals (38); next-generation compounds remain in early trials for other indications.
cGAS-STING inhibitors	Suppression of type-I IFN-driven SASP	Bone: preclinical only. Brain: preclinical only for aging-related neuroinflammation (22).	No aging-indication clinical trials; chronic pathway blockade may impair antiviral and antitumor immunity.
GLP-1 receptor agonists (semaglutide, liraglutide)	Anti-inflammatory and insulin-sensitising effects	Bone: meta-analytic BMD and turnover data suggest neutral to modest benefit, not fracture-powered (39). Brain: observational dementia-risk signals, with dementia-endpoint RCTs ongoing (40).	GI adverse effects, gallbladder disease, and sarcopenia risk with rapid weight loss, especially in frail osteoporotic older adults.
Microbiota-targeted therapies (probiotics, FMT)	Reduced endotoxaemia and SCFA restoration	Bone: small RCTs suggest modest BMD or bone-marker signals (41). Brain: small MCI/AD trials show inconsistent cognitive effects (42, 43).	Formulation heterogeneity, unsettled regulatory status, and FMT pathogen-transmission risk (44).
Multimodal exercise	Mechanical loading, metabolic improvement, and anti-inflammatory effects	Bone: established supportive benefit for musculoskeletal health. Brain: meta-analytic association with better cognitive performance in older adults (45).	Most evidence-supported foundational intervention, but not a stand-alone disease-modifying co-therapy.

Summarizes the current evidence for candidate dual-targeting interventions, together with their principal safety and translational limitations. The table is deliberately conservative: highest available evidence level is graded for each of the bone and brain endpoints, and no intervention is designated as clinically ready for the bone–brain comorbidity indication.

### 2.4 The mitochondria–cGAS-STING axis

The mitochondria–cGAS-STING axis is highlighted here because it is the single molecular node that will re-appear in Sections 3.2.3, 4.1.1, and 5.4.1 as a candidate unifying driver of skeletal, microglial, and systemic inflammaging. Dysfunctional mitochondria release mtDNA into the cytosol, activating the cytosolic DNA sensor cGAS; the STING–TBK1–IRF3 axis then induces type I interferon responses in parallel with NF-κB-driven pro-inflammatory transcription (29).

A pivotal study reported that pharmacological blockade of STING suppressed the pro-inflammatory secretory phenotype of senescent human cells and tissues and attenuated aging-associated inflammation in peripheral organs and in the brain of mice; in that model, cytosolic DNA released from dysfunctional mitochondria drove microglial cGAS activity and neurodegeneration-associated transcriptional states (22). These findings, however, remain preclinical: no cGAS or STING inhibitor has entered clinical trials for aging or neurodegeneration, and the essential physiological role of cGAS-STING in antiviral and antitumor immunity implies a fundamental safety trade-off that is not yet resolved (47). Complementary evidence indicates that partial mitochondrial outer-membrane permeabilization (“minority MOMP”) releases mtDNA via BAX/BAK macropores to drive SASP; *in vivo* MOMP inhibition reduced circulating inflammatory markers and improved murine healthspan (48). Whether this axis operates in the human aging skeleton and brain remains to be shown.

### 2.5 Perpetuating factors

#### 2.5.1 Mitochondrial dysfunction

Mitochondrial dysfunction—accumulation of mtDNA mutations, imbalance of fusion/fission dynamics, and declining mitophagy—represents one of the most critical endogenous drivers of inflammaging (49). It engages the cGAS-STING axis (Section 2.4) and NLRP3 inflammasome (Section 2.3) through mtDNA leakage and excessive ROS, respectively (50). The concept of “mitokines” (FGF21, GDF15) as circulating signals of mitochondrial stress has attracted attention as a candidate biomarker of systemic inflammaging (51); however, mitokine measurements are not yet standardized for clinical bone–brain risk stratification, and the causal contribution of these signals—as opposed to their status as bystander markers—remains uncertain.

#### 2.5.2 Gut dysbiosis

Age-related gut dysbiosis contributes to inflammaging through metabolic endotoxemia (translocation of LPS via a compromised intestinal barrier, driving TLR4–NF-κB activation in monocytes/macrophages) and through reduced production of anti-inflammatory short-chain fatty acids, particularly butyrate (52, 53). A widely cited longitudinal cohort reported that gut-microbiome “uniqueness” is associated with reduced all-cause mortality (54); however, this finding rests on a single cohort and has not yet been independently replicated in ethnically distinct populations, and its causal directionality is unresolved. Centenarian cohorts retain youth-associated microbial signatures (e.g., enrichment of Akkermansia muciniphila) associated with lower inflammatory markers (55). The therapeutic relevance of microbiota-targeted interventions for bone–brain comorbidity is critically appraised in Section 7.3.

#### 2.5.3 Immunosenescence

Immunosenescence and inflammaging are mutually reinforcing (56). Structural changes include thymic involution and reduced naive T-cell output, impaired B-cell affinity maturation, declined NK-cell cytotoxicity, and pro-inflammatory M1-skewed monocyte–macrophage polarization (57). The terminally differentiated CD8+ TEMRA subset expands in peripheral blood and constitutes a chronic cellular source of TNF-α and IFN-γ (58). Chronic cGAS-STING activation in aging immune cells drives type-I interferon transcription and represents a candidate nexus linking immunosenescence with inflammaging (57).

#### 2.5.4 Epigenetic remodeling

Epigenetic aging—global DNA hypomethylation with locus-specific hypermethylation, altered histone modifications, and heterochromatin loss—has been extensively reviewed elsewhere and is not re-elaborated here. The single element carried forward to Sections 3 and 4 is the progressive derepression of LINE-1 (L1) retrotransposons in senescent cells, which induces type I interferon production and amplifies SASP (59), and thereby links epigenetic aging to the cGAS-STING axis (Section 2.4).

Collectively, mitochondrial dysfunction, gut dysbiosis, immunosenescence, and epigenetic remodeling converge on the four molecular nodes summarized in Sections 2.1–2.4. In the remainder of the review, we do not re-describe these upstream signaling anatomies; we track how they are functionally re-engaged in bone (Section 3), brain (Section 4), and the bidirectional bone–brain axis (Section 5).

## 3 Inflammaging-mediated skeletal aging

### 3.1 Homeostatic bone remodeling: a brief foundation

This subsection provides only the minimum functional background required for Section 3.2; a comprehensive treatment is available elsewhere (60, 61). Bone remodeling maintains skeletal mechanical integrity through the spatiotemporally coupled activity of osteoblasts (OBs, derived from BM-MSCs and driven by BMP and Wnt/β-catenin signaling under RUNX2/Osterix control) and osteoclasts (OCs, derived from mononuclear phagocyte precursors under M-CSF and RANKL) within the basic multicellular unit (BMU) (62). Osteocytes—embedded terminally differentiated osteoblasts constituting ~90% of bone cells—are the principal source of RANKL and act as central hubs integrating mechanical and immune signals (20, 63).

The OPG/RANKL/RANK axis governs osteoclastogenesis. RANKL–RANK binding on osteoclast precursors recruits TRAF6 to activate NF-κB, MAPK, and c-Fos signaling, driving NFATc1-dependent osteoclast differentiation (64). OPG, secreted by osteoblasts and stromal cells, acts as a decoy receptor for RANKL, and the OPG/RANKL ratio is the core parameter controlling the resorption–formation balance (65). PTH, estrogen, mechanical loading, and inflammatory cytokines all modulate this ratio (20). The disease-relevant consequence—and the point at which inflammaging enters this circuit—is the sustained reduction of the OPG/RANKL ratio under chronic pro-inflammatory drive, discussed in Section 3.2.1.

### 3.2 Mechanisms by which chronic inflammation disrupts skeletal homeostasis

#### 3.2.1 Pro-inflammatory cytokine-driven osteoclast activation

Building on the SASP and NF-κB circuits summarized in Section 2, we focus here on how these signals are functionally re-engaged in bone. IL-1β, IL-6, TNF-α, and IL-17 are the principal inflammatory drivers of osteoclast activation, operating through two mechanisms: (i) upregulation of the RANKL/OPG ratio in osteoblasts and stromal cells, and (ii) direct NF-κB activation in osteoclast precursors (66, 67). IL-6 acts via classical membrane-bound IL-6Rα/gp130 and trans-signaling through soluble IL-6R (sIL-6R), whose circulating levels rise with age, to upregulate RANKL and suppress OPG (68). TNF-α promotes precursor differentiation and survival and sensitizes precursors to subthreshold RANKL concentrations (67). IL-1β synergizes with TNF-α to promote NFATc1 activation through RANKL-independent pathways (69). IL-17 acts through IL-17RA on osteoclast precursors (70). These mediators together form a self-reinforcing loop coupling inflammaging to age-related bone loss (67).

#### 3.2.2 Suppression of osteoblast function via impaired Wnt signaling

Osteoblast differentiation depends on canonical Wnt/β-catenin signaling: Wnt engagement of LRP5/6 stabilizes β-catenin, which activates RUNX2, OPG, and osteocalcin transcription (71). Under inflammaging, TNF-α upregulates the Wnt antagonist DKK1, and IL-1β and TNF-α induce SOST expression in osteocytes; the resulting rise in sclerostin, a potent Wnt inhibitor via LRP5/6 binding, suppresses osteogenesis (72). Age-associated ROS activate FOXO transcription factors that competitively sequester β-catenin (71); chronic NF-κB signaling promotes Smurf1/2-driven RUNX2 degradation (67); and IL-6–STAT3 indirectly suppresses osteoblast function through SOST upregulation (68). The convergent output is uncoupling of formation from resorption, and progressive net bone loss (73). **Sclerostin is a particularly instructive node:** it links skeletal Wnt inhibition to potential central-nervous-system Wnt disruption, an extension explored in Section 5.2.2.

#### 3.2.3 Toxification of the bone microenvironment by SASP factors

Senescent osteoblasts, osteocytes, endothelial cells, and bone-marrow stromal cells accumulate in aged bone tissue and secrete SASP components—high concentrations of IL-1α, IL-1β, IL-6, and MMPs (MMP-1, MMP-3, MMP-13)—that directly degrade type I collagen and compromise structural integrity (74, 75). Notably, osteocyte-derived SASP contains substantially elevated RANKL, driving paracrine amplification of osteoclast activation (76, 77). SASP signals propagate senescence through NF-κB activation in adjacent bone cells, with sustained mTORC1 activation amplifying translational output (69).

Evidence tier and controversy. Accumulating preclinical evidence positions osteocyte senescence as an important contributor to skeletal aging (76, 77). Definitive causal evidence in humans, however, remains limited and rests principally on murine conditional-clearance models. Senolytic strategies (Section 7.3) have demonstrated efficacy in animal studies (78), and a Phase 2 randomized trial of intermittent dasatinib plus quercetin (D+Q) in postmenopausal women (n = 60) reported an increase in the bone formation marker P1NP (+34%) and a modest gain in radial BMD (+2.7%), but these effects were confined to the subgroup with elevated baseline senescent-cell burden, and no cognitive-endpoint trial has yet been conducted (33). The critical appraisal of senolytics as a bone–brain intervention is deferred to Section 7.3.

### 3.3 Manifestations and consequences of skeletal aging

#### 3.3.1 Decline in BMD and deterioration of bone microarchitecture

Progressive BMD decline is the most direct macroscopic manifestation of skeletal aging. Peak bone mass is achieved around the third decade of life; women experience accelerated loss in the five to ten years following menopause, and men a more gradual decline. Both patterns are associated with elevated circulating IL-6 and TNF-α, suggesting inflammaging as a systemic driver of bone loss (79). Osteoporosis increases the risk of fragility fractures of the hip, vertebrae, and wrist, a major cause of disability and mortality in older populations (79).

HR-pQCT studies show characteristic age-related decreases in trabecular number, thinning, and loss of three-dimensional connectivity, alongside progressive cortical porosity and endosteal expansion; these changes correlate with circulating inflammatory markers (80). Accumulation of advanced glycation end products (AGEs) generates non-enzymatic collagen crosslinks that replace normal pyridinoline crosslinks, increasing matrix brittleness and reducing toughness (81). Age-related degeneration of the osteocyte network—reduced osteocyte density, disrupted dendritic connectivity, and perilacunar hypermineralization—impairs mechanosensing (63), and inflammaging-associated oxidative stress directly damages osteocyte mitochondria, promoting apoptosis and lacunar emptying (82). The quantitative contribution of inflammaging-driven microarchitectural change relative to sex-hormone loss, physical inactivity, and vitamin D status—the more clinically actionable drivers—has not been formally partitioned in human cohorts and represents an important gap (see Section 7.2).

#### 3.3.2 Adipogenic lineage bias of bone-marrow mesenchymal stem cells

With aging and inflammaging progression, BM-MSC differentiation shifts toward adipogenesis, producing bone-marrow adipose tissue (BMAT) accumulation (83). The mutual antagonism of adipogenic PPARγ and osteogenic RUNX2/Wnt/β-catenin signaling forms the molecular “seesaw” of MSC lineage fate (84). Pro-inflammatory signals activate NF-κB in MSCs, suppressing RUNX2 and enhancing PPARγ transcriptional activity (83). Age-associated ROS activate FOXO to divert β-catenin (71); epigenetic alterations further destabilize the osteo/adipo balance (83); and adipogenic microRNAs (e.g., miR-188) upregulate with age and target osteogenic signaling (85). Bone-marrow adipocytes establish a pro-inflammatory positive feedback through leptin secretion, persistently suppressing osteogenesis and promoting resorption (86).

#### 3.3.3 Formation of an inflammatory bone-marrow microenvironment

Single-cell transcriptomic analyses have revealed that aged skeletal stem cells (SSCs) in the bone marrow form an inflammatory degenerative niche characterized by systematic upregulation of inflammation-associated genes (74). Senescent cells accumulating with age continuously secrete SASP factors, converting the bone marrow into a chronically inflamed microenvironment (76). IL-17 signaling through IL-17RA promotes precursor proliferation and differentiation (70). M1-polarised macrophages, increased in aged bone marrow, sustain TNF-α, IL-1β, and IL-6 secretion, suppressing MSC osteogenesis and activating osteoclast precursors (70). Senescent osteocytes influence hematopoietic stem cell (HSC) lineage commitment through paracrine mechanisms (77), and persistent NLRP3 activation in aged stromal cells sustains IL-1β production (69).

##### 3.3.3.1 Balanced interpretation

The framing above—”pro-inflammatory bone-marrow niche”—simplifies a heterogeneous cellular landscape whose functional consequences may not be uniformly deleterious. Some SASP components support tissue remodeling and immune surveillance, and the boundary between adaptive and maladaptive stromal inflammation in the aged bone marrow is not sharply defined. The clinical significance of the “aged inflammatory niche” concept therefore requires validation through human single-cell datasets and prospective cohort studies before it can be operationalized as a therapeutic target (87).

**Figure 2:**
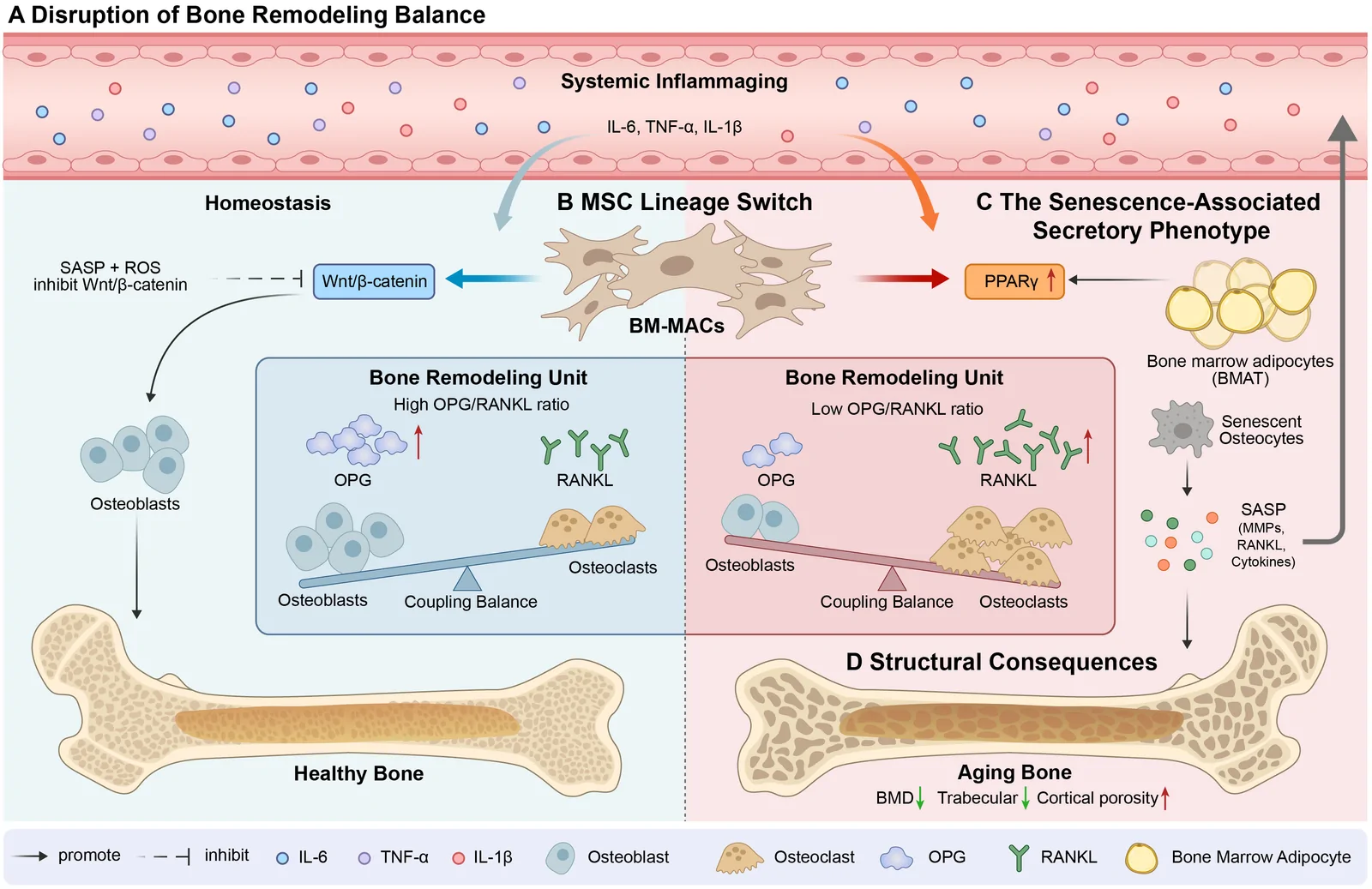
Mechanistic interplay between inflammaging and skeletal aging. **(A)** Systemic inflammaging exposes bone to IL-6, TNF-alpha, and IL-1beta, disrupting the balance between bone formation and resorption. **(B)** In the homeostatic state, Wnt/beta-catenin signaling supports osteoblast differentiation and a high OPG/RANKL ratio. **(C)** Under senescence-associated secretory phenotype (SASP) pressure, PPARgamma-driven marrow adipogenesis, senescent osteocytes, and SASP factors lower the OPG/RANKL ratio and increase osteoclast dominance. **(D)** These changes culminate in aging bone characterized by reduced BMD, trabecular deterioration, and increased cortical porosity.

Taken together, these multilevel mechanisms indicate that inflammaging remodels bone metabolic homeostasis through a pathological cascade—chronic pro-inflammatory cytokine networks, SASP-driven paracrine amplification, and consolidation of a pro-inflammatory bone-marrow microenvironment—that has been implicated in progressive BMD loss, microarchitectural deterioration, MSC adipogenic lineage bias, and coordinated decline of bone-marrow hematopoietic and osteogenic function. The relative causal weight of this cascade in human age-related osteoporosis, compared with sex-hormone loss and mechanical unloading, remains to be established. The bone-derived signals thus generated—reduced OCN, elevated SOST—propagate to the central nervous system, providing the biological substrate for the bone-to-brain arm of the bone–brain axis explored in Section 5. **Figure 2** illustrates the mechanistic interplay in schematic form.

## 4 Inflammaging-mediated cognitive impairment

### 4.1 Association between neuroinflammation and cognitive function

#### 4.1.1 Microglial activation and functional alterations

Microglia are the resident immune cells of the CNS and play a central role in maintaining brain homeostasis, clearing damaged tissue, and supporting synaptic remodeling (88). With advancing age, microglia progressively transition from a ramified resting state to a hyperactivated pro-inflammatory phenotype through a process termed “priming” (89).

Under acute injury, activated microglia can adopt a pro-repair phenotype secreting TGF-β, IL-4, IL-10, and neurotrophic factors (90). However, the chronic low-grade inflammation characteristic of inflammaging sustains microglia in a pro-inflammatory state, driving continuous secretion of TNF-α, IL-1β, IL-6, and reactive oxygen species (ROS) (21).

Preclinical data indicate that aged microglia exhibit progressive impairment of phagocytic function, DNA damage accumulation, mitochondrial dysfunction, and sustained upregulation of pro-inflammatory genes (91, 92). A subset of senescent microglia acquire the senescence-associated secretory phenotype (SASP), propagating inflammatory signals through paracrine mechanisms; in murine models, selective clearance of senescent glial cells markedly attenuates tau-dependent pathological progression (93). Single-cell transcriptomic studies have revealed profound spatiotemporal heterogeneity, with disease-associated microglia (DAM) and other state-specific subpopulations identified in neurodegenerative disease such as Alzheimer’s disease (AD) (94, 95).

##### 4.1.1.1 Controversy: microglial heterogeneity and the limits of binary framing

The pro-inflammatory framing used above simplifies a rapidly evolving landscape. Single-cell and spatial transcriptomic studies have delineated a continuum of context-dependent microglial states — homeostatic, DAM1/DAM2, IFN-responsive, MHC-II^high^, and lipid-associated — whose functional outputs are neither uniformly deleterious nor uniformly protective (94, 95). DAM signatures, in particular, may reflect an adaptive response to Aβ pathology rather than a purely pathogenic phenotype. References to “microglial activation” in this review should therefore be read as shorthand for a heterogeneous state space, not a homogeneous pro-inflammatory conversion.

#### 4.1.2 Mechanisms underlying blood–brain barrier integrity disruption

The blood-brain barrier (BBB) is formed by cerebral microvascular endothelial cells, pericytes, astrocytic endfeet, and the basement membrane, constituting the neurovascular unit (NVU) together with neurons (96). The BBB restricts non-selective entry of macromolecules and peripheral immune cells through precise regulation of endothelial tight junction proteins — claudin-5, occludin, and ZO-1 — providing neurons with immune-privileged protection (97).

Elevated circulating pro-inflammatory cytokines (IL-1β, TNF-α, IL-6) act directly on cerebral microvascular endothelial cells, activating NF-κB signaling and inducing MMP-2/MMP-9 secretion, which degrade tight junction proteins and basement-membrane collagen (98). With advancing age, pericyte density and function progressively decline, and this pericyte-dependent increase in BBB permeability has been reported in human studies as an early biomarker of cognitive decline (99). Astrocytes undergo reactive astrogliosis in chronic neuroinflammation, transitioning from a supportive to a pro-inflammatory phenotype (100). Excessive ROS directly disrupt the assembly of tight-junction complexes, further compromising barrier integrity (101). These mutually reinforcing mechanisms collectively elevate BBB permeability, permitting peripheral inflammatory signals, immune cells, and neurotoxic proteins to penetrate the brain parenchyma and thereby amplifying central neuroinflammation (97).

### 4.2 Transmission of peripheral inflammation to the central nervous system

#### 4.2.1 Routes of inflammatory cytokine entry into the brain

Transmission of peripheral systemic inflammation to the CNS involves multiple parallel pathways (102). Structural leakage of the aging BBB permits small cytokine molecules to penetrate directly via the paracellular route; peripheral pro-inflammatory cytokines can additionally bind IL-1R and TNFR on BBB endothelial cells, transmitting inflammatory signals through transcellular signal transduction (98). Chronic low-grade inflammation upregulates the adhesion molecules VCAM-1 and ICAM-1, enhancing peripheral immune cell transmigration (101).

The vagus nerve serves as a critical neuroanatomical conduit for transmitting peripheral inflammatory signals to the CNS, mediating a body–brain circuit capable of sensing and modulating systemic inflammation (103). Vagal sensory neurons of the nodose ganglion express multiple cytokine receptors, detecting peripheral IL-1β, IL-6, and TNF-α signals and relaying them to the nucleus tractus solitarius (NTS); distinct sensory neuron subtypes selectively encode different cytokines, and preclinical evidence indicates that chronic inflammation disrupts this specificity, reducing the precision of central regulation of inflammatory signaling (104). Through projections to the hypothalamus and prefrontal cortex, the NTS translates peripheral inflammatory signals into a brain-wide pro-inflammatory activation state (103). The efferent cholinergic anti-inflammatory pathway (CAP) of the vagus nerve suppresses cytokine production via α7 nicotinic acetylcholine receptors (α7nAChR) on peripheral immune cells; the efficacy of this pathway is attenuated in the context of inflammaging (105).

#### 4.2.2 Brain infiltration by peripheral immune cells

Under physiological conditions, entry of peripheral monocytes, T lymphocytes, and other immune cells into the brain parenchyma is extremely limited; CNS immune surveillance is primarily maintained by resident microglia (102). Under the combined influence of chronic inflammation and BBB disruption, brain infiltration by peripheral immune cells has been shown, principally in preclinical models and supported by post-mortem human observations, to increase markedly (106).

Peripheral monocytes are driven by CCL2/MCP-1 chemokine gradients across the disrupted BBB and differentiate in the parenchyma into pro-inflammatory macrophages that synergize with resident microglia in releasing neurotoxic mediators. Age-associated expansion of TEMRA (terminally differentiated effector memory re-expressing CD45RA) CD8^+^ T cells further exacerbates the imbalance between pro- and anti-inflammatory immunity (106).The superimposition of peripheral immune infiltration on aberrant activation of the local innate immune system collectively provides the cellular foundation for perpetuation of neuroinflammation and progressive cognitive dysfunction in inflammaging.

### 4.3 Inflammatory mechanisms in neurodegenerative disease

#### 4.3.1 Inflammatory facilitation of Aβ deposition and tau phosphorylation in Alzheimer’s disease

Neuroinflammation has been proposed, principally on the basis of preclinical evidence, to play a facilitating role in the formation and propagation of the two hallmark pathological features of AD — extracellular Aβ plaque deposition and intracellular tau hyperphosphorylation — with these processes forming a bidirectional cycle (107). Aβ oligomers and fibrillar aggregates function as endogenous damage-associated molecular patterns (DAMPs), interacting with pattern recognition receptors on microglial surfaces (TLR2, TLR4, TLR6, CD36, RAGE) to activate downstream NF-κB signaling and prime the NLRP3 inflammasome (36). Phagocytosis of Aβ fibers increases lysosomal membrane permeability, allowing cathepsin B to activate NLRP3, driving caspase-1–mediated cleavage of pro-IL-1β and pro-IL-18 (36). Sustained IL-1β further suppresses microglial phagocytic clearance of Aβ, establishing a positive feedback loop of “Aβ accumulation → inflammatory activation → impaired Aβ clearance” (108).

In murine models, tau aggregates similarly activate the NLRP3 inflammasome via a lysosomal-disruption mechanism, upregulating the tau kinases GSK-3β and CDK5 and promoting aberrant tau hyperphosphorylation (37). Genetic or pharmacological inhibition of NLRP3 concomitantly reduces Aβ plaque burden and tau phosphorylation and improves cognitive function in these models, providing preclinical causal evidence that NLRP3 mechanistically links Aβ and tau pathologies (37). Extracellular Aβ-ASC complexes can act as seeds propagating Aβ aggregation in surrounding neurons (36). NF-κB serves as a central inflammatory nexus linking Aβ and tau pathologies (109). Direct human causal evidence remains restricted to associative biomarker studies and small early-phase trials of NLRP3-pathway modulators.

#### 4.3.2 Impairment of hippocampal neurogenesis and decline of synaptic plasticity

Adult hippocampal neurogenesis (AHN) occurs primarily in the subgranular zone (SGZ) of the dentate gyrus; newly generated granule neurons play an indispensable role in contextual memory encoding and pattern separation (110). Chronic neuroinflammation driven by inflammaging has been implicated in the progressive decline of AHN, principally on the basis of rodent studies (111). In these models, IL-1β reduces neural stem cell proliferation via NF-κB and upregulates cell-cycle inhibitors to induce premature progenitor senescence; TNF-α induces progenitor apoptosis and downregulates BDNF and VEGF; and IL-6 skews neural progenitor differentiation toward astroglia via JAK–STAT3, collectively reducing the supply of functional newborn neurons (110). In humans, post-mortem studies report that the density of neurogenesis markers in the dentate gyrus (Nestin^+^ neural stem cells, DCX^+^ neuroblasts) is lower in AD than in cognitively healthy controls, although the persistence and quantification of AHN in adult humans remain areas of active debate (112).

Decline of synaptic plasticity is the cellular electrophysiological substrate of cognitive impairment. Long-term potentiation (LTP) has been shown, largely in rodent slice preparations, to be impaired at multiple targets by neuroinflammation (113). IL-1β disrupts AMPA-receptor trafficking and NMDA-receptor function at excitatory synapses; TNF-α reduces dendritic spine density by promoting internalization of surface AMPA receptors and downregulating PSD-95. Attenuation of BDNF–TrkB signaling further contributes to progressive cognitive decline (113).

#### 4.3.3 Oxidative stress and neuronal apoptosis

Oxidative stress and neuroinflammation form a bidirectional amplification loop that has already been described in Section 2 and is not re-elaborated here. In neurons, this loop culminates in mitochondrial-permeability-transition-pore–mediated cytochrome c release and caspase-3/7-driven intrinsic apoptosis, and in TNF-α–TNFR1–driven extrinsic apoptosis and necroptosis (114). The functionally distinctive feature in the aging brain is the coincident collapse of neuronal antioxidant reserves and the compounding effect of mtDNA release via the cGAS-STING pathway on microglial priming (22); the latter has been proposed as a therapeutically tractable driver of age-related neuroinflammation in murine models, but human validation and inhibitor development remain at an early stage.

**Figure 3:**
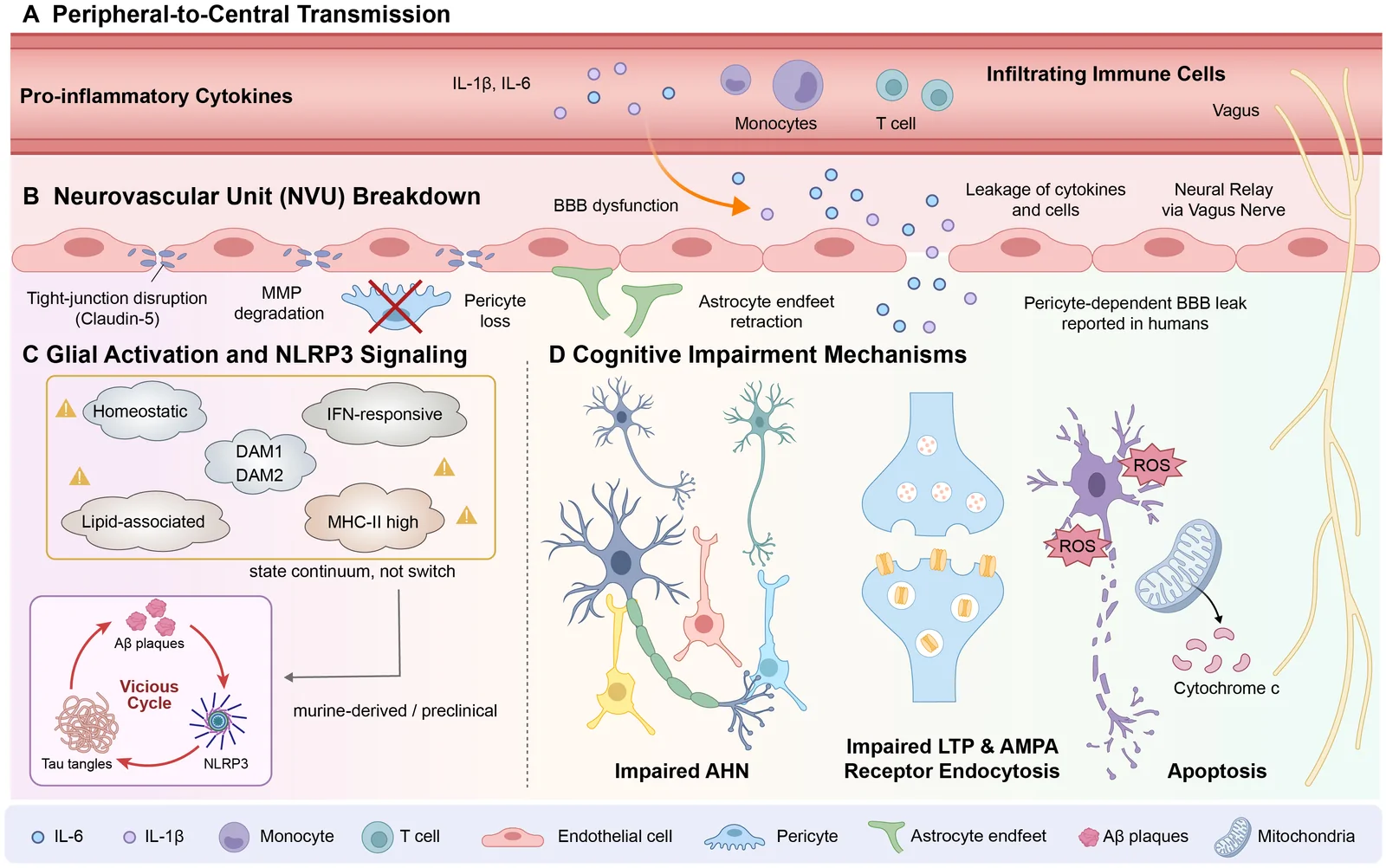
Routes linking systemic inflammaging to cognitive impairment through neuroimmune and neurovascular mechanisms. **(A)** Peripheral inflammatory cytokines and infiltrating immune cells, including monocytes and T cells, communicate with the CNS through BBB disruption and neural relay via the vagus nerve. **(B)** Neurovascular-unit breakdown includes tight-junction disruption, MMP-mediated matrix degradation, pericyte loss, astrocyte-endfeet retraction, and pericyte-dependent BBB leak reported in humans. **(C)** Glial activation is depicted as a state continuum, including homeostatic, DAM1/DAM2, IFN-responsive, lipid-associated, and MHC-II-high states, rather than as a binary switch; the NLRP3-A beta/tau cycle remains mainly preclinical. **(D)** Cognitive impairment mechanisms include impaired adult hippocampal neurogenesis, impaired LTP and AMPA-receptor endocytosis, oxidative stress, mitochondrial cytochrome-c release, and apoptosis.

Taken together, inflammaging is proposed to impair cognitive function and drive the initiation and progression of neurodegenerative disease through a multilayered set of mechanisms — sustained microglial activation (21), progressive BBB disruption (97, 99), multi-route transmission of peripheral inflammatory signals to the CNS (102, 103), peripheral immune infiltration (106), the NLRP3–Aβ/tau bidirectional cycle (36, 37), impaired hippocampal neurogenesis (110, 112), declining synaptic plasticity (113), and oxidative-stress–driven neuronal apoptosis (114). The strength of causal evidence varies across these axes: several are supported by robust preclinical mechanistic data but limited human corroboration, an evidence gap addressed explicitly in Section 7.2. **Figure 3** summarizes the logical chain by which peripheral inflammation contributes to central cognitive impairment.

## 5 The bone–brain axis: a bidirectional pathway linking skeletal aging to cognitive impairment

### 5.1 Conceptual basis and evidence for the bone–brain axis

#### 5.1.1 Conceptual framework

Beyond its structural role, bone functions as an endocrine organ by secreting bioactive molecules — including osteocalcin (OCN), sclerostin (SOST), and fibroblast growth factor 23 (FGF23) — that regulate distant target organs, including the brain (115). Conversely, the central nervous system (CNS) exerts reciprocal control over bone metabolism through sympathetic neural outflow and neuropeptide signaling. This bidirectional interactive network between bone and brain has been conceptualized as the bone–brain axis (BBA) (115). Its overall quantitative contribution to age-related cognitive and skeletal decline, relative to other systemic drivers, has not yet been rigorously partitioned in human studies.

The BBA is proposed to operate through three core pathways: bone-derived molecules traverse the blood-brain barrier (BBB) via the systemic circulation to act on neurons and glial cells; the CNS regulates bone remodeling through autonomic efferent signaling; and the bone marrow, as the primary site of hematopoiesis and immune cell generation, influences neurological function through alterations in its inflammatory microenvironment. These three pathways may converge and amplify one another in aging, providing a plausible pathological basis for the co-progression of skeletal deterioration and cognitive decline (115).

#### 5.1.2 Epidemiological evidence linking low BMD to cognitive impairment

Low bone mineral density (BMD) and cognitive impairment are highly co-prevalent in older adults, and accumulating epidemiological evidence supports an association independent of shared risk factors such as age, cardiovascular disease, and metabolic disorders. The Rotterdam Study (n = 3,401; follow-up 11 years) demonstrated that reduced femoral-neck BMD was significantly associated with increased risk of all-cause dementia and AD (HR per SD = 1.25; 95% CI 1.02–1.53) (7).

A meta-analysis incorporating three large cohort studies confirmed that lower baseline BMD remained significantly associated with dementia incidence after adjustment for confounders (116). A separate meta-analysis (10 studies; n = 9,872) found that patients with cognitive impairment had a 1.56-fold greater risk of osteoporosis than controls (RR = 1.56; 95% CI 1.30–1.87), with AD patients exhibiting an even higher risk (RR = 1.70) (6).

Reductions in BMD have been reported to co-occur with decreased plasma Aβ42/40 ratios and elevated central amyloid burden, suggesting shared biological drivers (117). Prospective data from the Canadian Multicentre Osteoporosis Study (CaMOS) further demonstrated that cognitive decline in women is positively associated with accelerated bone loss and increased fracture risk (118). Nevertheless, these associations are dominated by observational designs with limited adjustment for shared upstream drivers (physical activity, vitamin D and K status, sex-hormone milieu), and adequately powered Mendelian-randomisation analyses of the BMD–cognition relationship remain scarce; BMD should therefore be regarded as a candidate rather than validated biomarker of cognitive risk (119).

### 5.2 Effects of bone-derived signaling molecules on the brain

#### 5.2.1 Osteocalcin: promoting neurogenesis and improving memory

Osteocalcin (OCN) is a 49-amino-acid γ-carboxyglutamate protein synthesized by osteoblasts that enters the circulation in its undercarboxylated form (ucOCN). In murine models, OCN has been reported to cross the BBB and to bind GPR158 and GPR37 on neurons in the hippocampal CA3 region and ventral tegmental area (VTA) (120).

Also in murine models, OCN acting through GPR158 has been reported to enhance tryptophan- and tyrosine-hydroxylase activity, thereby promoting serotonin and dopamine synthesis, and to activate an IP3/BDNF pathway regulating spatial learning and memory (120); whether these mechanisms operate in humans remains uncertain (see Controversy statement below).

With respect to neurogenesis, preclinical evidence suggests that OCN upregulates BDNF and VEGF expression via a PI3K/Akt/CREB axis, promoting neural stem cell proliferation and differentiation in the subgranular zone (SGZ) of the dentate gyrus and enhancing contextual memory encoding (121). In AD mouse models, OCN supplementation has been reported to reduce hippocampal amyloid-β (Aβ) plaque burden and to improve brain energy metabolism by upregulating neuroglial glycolysis, thereby ameliorating cognitive deficits (122).

Human evidence is primarily associative. Cross-sectional studies report that plasma and CSF OCN correlate with AD core biomarkers (Aβ, tau) and cognitive scores, with OCN declining as AD advances (123). Mendelian-randomisation analyses have provided genetic evidence consistent with a protective effect of OCN against AD (124), and aerobic exercise elevates circulating OCN, offering a partial biological framework for the cognitive benefits of physical activity (125).

Controversy: Translational uncertainty of osteocalcin-mediated neuroprotection. The proposed role of OCN as a bone-derived neurotrophic hormone rests principally on murine studies. This model has been challenged by independent groups reporting that *Bglap*/*Bglap2*-deficient mice generated on distinct genetic backgrounds do not reproduce the reported metabolic and cognitive phenotypes (51, 52). In humans, associations between circulating OCN and cognitive performance are inconsistent across cohorts and are confounded by vitamin K status, physical activity, and bone-turnover rate. The clinical significance of OCN–GPR158 signaling should therefore be regarded as an active hypothesis requiring rigorous prospective and mechanistic validation, rather than an established therapeutic axis.

#### 5.2.2 Sclerostin: adverse effects on cognitive function

Sclerostin is a glycoprotein synthesized by osteocytes and encoded by the *SOST* gene. It inhibits osteoblast activity and promotes bone resorption by binding LRP5/6 co-receptors and antagonizing Wnt/β-catenin signaling. *SOST* expression is markedly upregulated in senescent osteocytes, and circulating sclerostin rises progressively with age, correlating inversely with BMD (126).

In preclinical models, pathologically elevated osteocyte-derived sclerostin has been reported to traverse the BBB and to disrupt neuroprotective Wnt/β-catenin signaling, impairing synaptic plasticity and spatial memory. Mechanistically, sclerostin has been proposed to promote Aβ production via the β-catenin–β-secretase 1 (BACE1) axis; in an AD mouse model, bone-specific SOST overexpression accelerated Aβ accumulation and cognitive decline, whereas functional sclerostin blockade ameliorated these phenotypes (127).

Clinical data indicate that plasma sclerostin in cognitively unimpaired older adults correlates significantly with brain Aβ-PET positivity (ρ = 0.321, P = 0.001); Aβ-positive individuals exhibit higher plasma SOST than Aβ-negative controls (71.49 vs. 56.51 pmol/L, P < 0.01), suggesting that sclerostin may serve as a plasma biomarker for preclinical AD (128). Direct human evidence for sclerostin BBB penetration and CNS action, however, is currently lacking; whether peripheral SOST elevations translate to central effects, or merely serve as a shared marker of underlying osteocyte senescence, is unresolved. Romosozumab, an anti-sclerostin monoclonal antibody approved for osteoporosis, should therefore be considered a mechanistic test case rather than a candidate bone–brain co-therapy unless dedicated cognitive-endpoint studies show benefit.

#### 5.2.3 FGF23: an emerging role in the bone–brain axis

FGF23, primarily secreted by osteocytes, is the central endocrine regulator of phosphate metabolism and vitamin D homeostasis, with circulating levels rising markedly in impaired renal function and abnormal bone mineralization (129).

Adverse neurological effects reported for FGF23 are mediated principally through cerebrovascular injury: FGF23 binds FGFR-2 and FGFR-3 on vascular endothelial cells in an α-Klotho–independent manner, inducing vascular calcification and atherosclerosis, and thereby contributing to white-matter hyperintensities and subcortical microinfarcts that indirectly impair cognitive function (129). Excess FGF23 additionally suppresses 1,25-dihydroxyvitamin De_3_ synthesis, and vitamin D deficiency is an independent cognitive risk factor (53).

The translational interpretation of FGF23 as a bone-derived driver of cognitive decline is nevertheless constrained. Most human data linking FGF23 to cognitive outcomes derive from chronic-kidney-disease (CKD) cohorts, in which uraemic toxins, mineral–bone disorder, and vascular co-morbidities are potent confounders. Whether FGF23 acts as an independent bone–brain endocrine effector, or serves largely as a biomarker of coincident renovascular pathology, has not yet been dissected in non-CKD aging populations.

### 5.3 Neurogenic signals regulating bone metabolism

#### 5.3.1 Sympathetic nervous system regulation of bone remodeling

The sympathetic nervous system (SNS) constitutes the best-characterized efferent pathway through which the CNS controls bone metabolism. Osteoblasts abundantly express β2-adrenergic receptors (β2-AR), and norepinephrine (NE) released from sympathetic terminals activates the cAMP/PKA/ATF4 axis to suppress osteoblast proliferation while upregulating RANKL and inhibiting OPG secretion, thereby promoting osteoclast formation and net bone loss (130). The negative regulatory role of the SNS in bone metabolism is supported mainly by rodent β-adrenergic manipulation studies and disease-specific models. Human evidence is largely observational, including associations between β-blocker exposure and fracture risk, and is not sufficient to establish a causal neurogenic mechanism in skeletal aging.

In murine models of aging, persistently elevated central sympathetic tone suppresses bone formation and activates bone resorption via β2-AR signaling, providing a plausible neurological mechanism for age-related progressive bone loss (130). Osteocytes are directly innervated by sympathetic nerves; β2-AR activation has been reported to promote release of osteocyte-derived extracellular vesicles containing bone-degrading enzymes, mediating perilacunar bone resorption (131). The extent to which this pathway quantitatively contributes to human age-related osteoporosis, relative to endocrine and mechanical-loading drivers, remains unresolved.

The hypothalamus orchestrates SNS output to bone by integrating signals from leptin, serotonin, and other regulators, constituting a hypothalamus-SNS-skeleton regulatory circuit (132). Under inflammaging conditions, hypothalamic neuroinflammation is proposed to disrupt this circuit, contributing to pathologically elevated sympathetic tone that may simultaneously accelerate bone loss and impair emotional and cognitive regulation (133). This proposed link remains principally preclinical and should be tested in human longitudinal cohorts with synchronized autonomic, skeletal, and cognitive measures.

#### 5.3.2 Effects of neuropeptides on bone cells

Bone tissue is densely innervated by sensory and autonomic nerve fibers, and neuropeptides released from these terminals regulate bone metabolism by binding to receptors on bone cells, constituting a critical component of the neuro-skeletal signaling network.

Neuropeptide Y (NPY) exerts multilevel regulation through Y1 and Y2 receptor subtypes. Centrally, hypothalamic Y2-receptor activation suppresses bone formation; locally, Y1-receptor signaling modulates osteoblast differentiation and osteoclast function. Germline deletion of Y1 receptors increases bone formation, and elevated NPY levels are positively correlated with bone loss following ovarian insufficiency (134). Gut dysbiosis can exacerbate postmenopausal osteoporosis through upregulation of NPY signaling, suggesting multidimensional roles for NPY within the bone–brain-gut axis (135).

Vasoactive intestinal peptide (VIP) exerts dual pro-osteogenic and anti-osteoclastogenic effects through VPAC1 and VPAC2 receptors. VIP downregulates NFATc1 and osteoclast-differentiation genes including CSF1R and TNFRSF11A, reducing mature osteoclast numbers and bone-resorptive activity (136). VIP additionally promotes osteogenic differentiation of mesenchymal stem cells via VPAC1 (137).

Calcitonin gene-related peptide (CGRP) and other sensory neuropeptides promote bone formation by activating osteoblast receptors and regulating bone-marrow vascularization and local immune responses. Autonomic dysfunction associated with inflammaging is proposed to disrupt these neuropeptide networks, potentially driving co-progression of impaired bone metabolism and neurological dysfunction (138).

### 5.4 Inflammaging as the central amplifier of the bone–brain axis

#### 5.4.1 Concurrent damage to the skeletal and nervous systems by chronic inflammation

The chronic low-grade inflammatory microenvironment of inflammaging is proposed to exert concurrent damaging effects on both the skeletal and nervous systems, constituting a pathological basis of bone-cognitive comorbidity in older adults. In bone, TNF-α, IL-1β, and IL-6 activate the RANKL/OPG axis, promoting osteoclast differentiation while suppressing osteoblast function and resulting in progressive bone loss (139). In the CNS, the same cytokines activate microglia and astrocytes, disrupt the BBB, and promote peripheral immune-cell infiltration, establishing persistent neuroinflammation (140).

IL-6 serves as a central inflammatory mediator in the BBA: in bone metabolism it activates osteoclasts and promotes osteoporosis; in the nervous system it suppresses adult hippocampal neurogenesis via JAK-STAT3 (110). TNF-α exerts both bone-catabolic and neurotoxic activities, simultaneously promoting osteoclast differentiation and inducing synaptic plasticity impairment and neuronal apoptosis (140).

Oxidative stress is an important mechanism through which inflammaging concurrently damages bone and brain: elevated ROS impair osteoblast DNA and mitochondrial function, accelerating osteoblast apoptosis, and contribute to neurodegeneration through lipid peroxidation and mitochondrial dysfunction (141).

Cellular senescence and the SASP constitute a molecular link connecting bidirectional bone–brain injury. Senescent osteoblasts and osteocytes continuously release IL-6, IL-1β, and matrix metalloproteinases (MMPs) via the SASP, locally exacerbating osteoporosis while activating CNS-resident immune cells via the systemic circulation (142).

The NLRP3 inflammasome plays a critical role in concurrent BBA injury: NLRP3-derived IL-1β promotes RANKL-dependent osteoclast differentiation, aggravating osteoporosis (35), while in the CNS NLRP3 activation drives Aβ accumulation, tau hyperphosphorylation, and hippocampal neuronal apoptosis (36).

The mitochondria–cGAS-STING axis has, in addition, been proposed as a therapeutically tractable driver of age-related neuroinflammation in murine models (22); human validation and clinical inhibitor development remain at an early stage, and the analogous role of cGAS-STING signaling in age-related bone loss rests on limited preclinical data. These caveats notwithstanding, the axis is discussed here because it is one of the few molecular circuits invoked simultaneously in the aging bone and brain literatures.

Inflammaging additionally disrupts the hypothalamic-pituitary-gonadal axis, resulting in decreased sex hormone levels that concurrently accelerate osteoporosis and attenuate neuroprotective mechanisms (143). Chronic inflammation induces excess FGF23 secretion, suppressing 1,25-dihydroxyvitamin D_3_ synthesis and further reinforcing co-injury along the BBA (129).

#### 5.4.2 Mutual reinforcement between the bone marrow inflammatory microenvironment and neuroinflammation

The bone marrow serves as a central hub for the mutual reinforcement of BBA pathology under inflammaging conditions. The aging bone-marrow microenvironment undergoes multidimensional pathological remodeling: hematopoietic stem cells (HSCs) demonstrate progressive functional decline and myeloid skewing, generating a greater proportion of pro-inflammatory monocytes; bone-marrow adipogenesis expands, amplifying local inflammaging through paracrine secretion of IL-6 and TNF-α; and the osteogenic differentiation capacity of mesenchymal stem cells is markedly diminished (144).

Bone-marrow-derived inflammatory monocytes represent an important peripheral source of neuroinflammation. Myeloid skewing increases the number and pro-inflammatory activity of circulating monocytes; following BBB disruption, these cells are recruited to the brain parenchyma along CCL2/MCP-1 gradients and differentiate into potently pro-inflammatory macrophages that synergize with resident microglia to amplify neuroinflammation (21).

A bidirectional positive feedback loop has been proposed between bone marrow and CNS neuroinflammation: pro-inflammatory cytokines from bone-marrow inflammaging enter the brain via the systemic circulation, activating microglia and damaging the BBB (97); conversely, CNS neuroinflammatory signals feedback to regulate bone-marrow hematopoiesis through the autonomic nervous system, establishing a bone-marrow–brain neuroimmune axis (144). Direct *in-vivo* evidence for the operation of this loop in humans is currently limited, and it should be regarded as a mechanistically plausible framework rather than an established causal circuit (145).

The accumulation of SASP-expressing cells in the aging bone marrow may exert pathological effects across tissue boundaries. Senescent osteoprogenitors, HSCs, and stromal cells release pro-inflammatory mediators via the SASP that both damage the local osteogenic microenvironment and superimpose upon SASP signals from CNS-resident senescent microglia, producing a synergistic amplification of systemic and central aging-associated inflammation (146).

Concurrent upregulation of osteocyte-derived SOST and decreased OCN secretion may attenuate Wnt signaling–mediated neuroprotection and neurotrophic support in the brain (127). Excess FGF23 release could further accelerate cognitive decline through cerebrovascular pathways (127).

**Figure 4:**
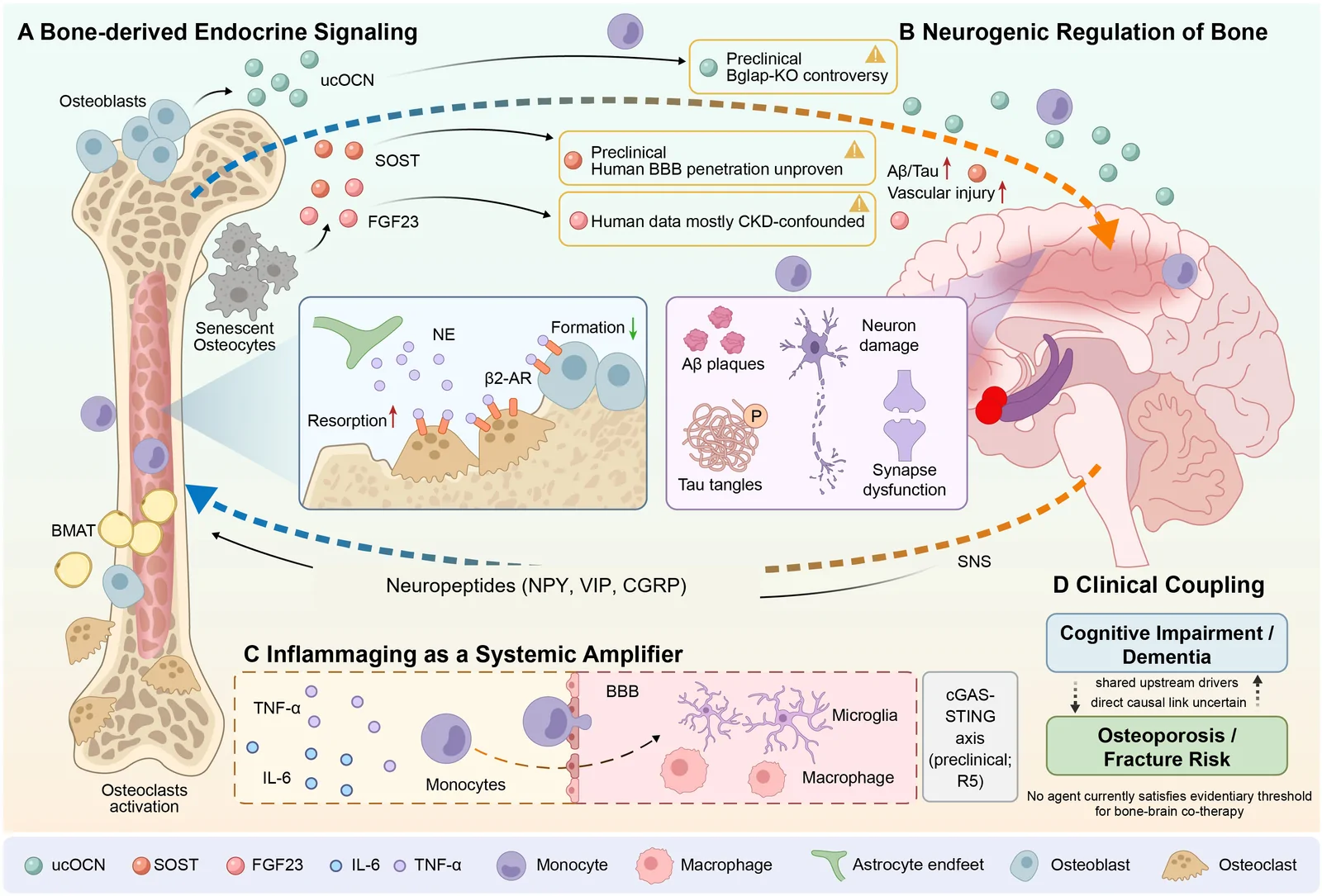
Bone–brain coupling under inflammaging with explicit evidence-boundary annotations. **(A)** Bone-derived endocrine signaling includes ucOCN, SOST, and FGF23, but their central relevance is limited by preclinical Bglap-KO controversy, unproven human BBB penetration for SOST, and CKD confounding for FGF23. **(B)** Neurogenic regulation of bone occurs through SNS and neuropeptide pathways that can increase resorption and reduce formation, but human causal evidence remains incomplete. **(C)** Inflammaging acts as a systemic amplifier through IL-6, TNF-alpha, monocytes, BBB disruption, microglia, macrophages, and the cGAS-STING axis, which remains preclinical in this context. **(D)** Clinical coupling between cognitive impairment/dementia and osteoporosis/fracture risk is framed as shared upstream drivers with uncertain direct causality; no agent currently satisfies the evidentiary threshold for bone–brain co-therapy.

**Figure 4** illustrates the pathways through which inflammaging acts as an amplifier connecting bone and brain. In summary, inflammaging is proposed to link skeletal aging and cognitive decline as a mutually reinforcing aging comorbidity through (i) concurrent pro-inflammatory cytokine-mediated injury to bone and CNS, (ii) bidirectional reinforcement between the bone-marrow inflammatory microenvironment and neuroinflammation, and (iii) pathological remodeling of bone-derived signaling molecules (decreased OCN, elevated SOST, elevated FGF23). Because the strongest current evidence for each of these limbs is derived from preclinical models, translation of this integrative framework into clinical practice requires dedicated human validation studies, as discussed in Section 7.2 (115).

## 6 Convergence of common pathological mechanisms

### 6.1 Mitochondrial dysfunction and energy metabolism

Mitochondria are the primary sites of cellular oxidative phosphorylation, and age-related mitochondrial deterioration represents a shared pathological driver of both osteoporosis and neurodegenerative disease. In both conditions, mtDNA mutation accumulation, dysregulation of mitochondrial dynamics, and impaired mitophagy converge as common substrates (147).

In bone, studies using the PolgA-mut/mut mtDNA-mutator mouse model have shown that mitochondrial respiratory-chain deficiency in osteoblasts and osteoclasts is associated with reduced bone-formation rate, decreased osteoblast density, increased osteoclast activity, and accelerated bone loss (147). In murine models, oxidative-stress-driven DRP1 upregulation in osteoblasts promotes excessive mitochondrial fission; pharmacological or genetic DRP1 inhibition restores mitochondrial function, alkaline phosphatase activity, and bone-nodule formation (148). PGC-1 alpha, via transcriptional activation of NRF-1, NRF-2, and TFAM, drives mtDNA replication and respiratory-chain assembly (41). PINK1/Parkin-mediated mitophagy is likewise essential; PINK1 deletion impairs mitochondrial homeostasis and exacerbates ovariectomy-induced bone loss in mice (149).

In the nervous system, loss-of-function mutations in PINK1 or Parkin lead to accumulation of dysfunctional mitochondria and dopaminergic neuronal death, representing a core mechanism in Parkinson disease (PD) (149). Mitochondrial dysfunction-induced energy failure is similarly implicated in AD synaptic-plasticity deficits (150).

NAD+ declines systemically with aging and is closely linked to mitochondrial deterioration and neurodegeneration. Nicotinamide mononucleotide (NMN), a direct NAD+ precursor, has attenuated aging-associated bone loss and neurodegenerative pathology in preclinical models, and a randomized placebo-controlled trial confirmed that NMN supplementation raises blood NAD+ and improves walking speed in older adults (42, 151).

The NAD+ - Sirtuin - PGC-1 alpha axis is therefore proposed as a candidate common therapeutic target for age-related bone and brain pathology; direct dual-endpoint clinical validation, however, is not yet available.

### 6.2 Systemic damage from oxidative stress

Oxidative stress - the imbalance in which ROS production exceeds antioxidant defence - occupies a central position in the pathogenesis of both osteoporosis and neurodegenerative disease and couples these pathological trajectories through systemic low-grade inflammation (152).

The upstream biology of ROS generation, NADPH-oxidase activation, and the mitochondrial contribution to oxidative injury has been described in Sections 2.2 and 6.1 and is not re-elaborated here. Below we retain only the elements specifically relevant to concurrent bone and brain injury.

In bone, elevated oxidative-stress biomarkers (malondialdehyde, 8-OHdG) correlate inversely with BMD, and the oxidative damage associated with postmenopausal estrogen deficiency is a central mechanism of postmenopausal osteoporosis (153).

ROS activates NF-kB and MAPK signaling to upregulate RANKL while suppressing OPG, raising the RANKL/OPG ratio and accelerating osteoclastogenesis (43). In osteoblasts and osteocytes, Nrf2 activation induces cytoprotective genes (HO-1, NQO1, GCLC) that maintain redox homeostasis; inhibition of Nrf2 degradation ameliorates bone loss driven by 1,25-dihydroxyvitamin D deficiency (43, 154). Nrf2 also protects osteoblasts from ferroptosis via the Keap1/Nrf2/SLC7A11/GPX4 pathway (155).

#### 6.2.1 Controversy: the bidirectional role of Nrf2 in bone

While the studies above position Nrf2 activation as cytoprotective in bone cells, other reports demonstrate that excessive or sustained Nrf2 activation may simultaneously impair both osteoblast and osteoclast differentiation, and that Keap1 loss-of-function models can develop a paradoxical low-bone-mass phenotype (154). The therapeutic implication is that the dose-response relationship for Nrf2-targeted interventions in bone is likely to be non-monotonic, and simple “the more Nrf2 the better” strategies are unlikely to succeed.

In the CNS, the brain is particularly vulnerable to oxidative damage (high oxygen consumption, abundant polyunsaturated fatty acids, limited antioxidant capacity). Excess ROS damages mitochondrial and nuclear DNA, promotes Abeta aggregation, induces tau hyperphosphorylation, and activates NF-kB-driven microglial neuroinflammation - features documented in both AD and PD (49). A positive-feedback loop between ROS and chronic inflammation drives further RANKL-dependent bone resorption and amplifies microglial neuroinflammation (49, 152). Together with the mitochondrial dysfunction described in Section 6.1, oxidative stress and neuroinflammation constitute a mutually reinforcing convergence node in bone–brain co-pathology; the relative causal weight of each limb, however, remains difficult to partition in human studies.

### 6.3 The gut–bone–brain axis

The gut microbiota regulates both bone metabolism and CNS function through bidirectional communication involving vagal afferents, gut-derived neurotransmitters, the hypothalamic-pituitary-adrenal (HPA) axis, and microbial metabolites. The “gut–bone–brain axis” framework offers a plausible dimension of shared pathology in osteoporosis and neurodegenerative disease (148).

In bone, glucocorticoid-induced osteoporosis models demonstrate that dysbiosis and compromised intestinal barrier function permit LPS translocation, triggering TLR4/NF-kB-dependent systemic low-grade inflammation that promotes osteoclastogenesis via RANKL/OPG (41). Short-chain fatty acids (SCFAs; acetate, propionate, butyrate) produced by microbial fiber fermentation inhibit osteoclast differentiation and promote osteogenic activity in preclinical models (41).

With respect to the CNS, SCFAs cross the BBB and modulate neuroinflammation via GPR41/GPR43 signaling, maintain BBB integrity, and influence neurotransmitter synthesis (149). In AD, gut microbiota alpha-diversity is reduced, with decreased SCFA-producing taxa and increased pro-inflammatory genera (e.g. *Desulfovibrio*, *Prevotellaceae*); systematic reviews and meta-analyses confirm compositional alterations across the AD spectrum, with dysbiosis correlating with cognitive decline (42, 150). Butyrate additionally acts through HDAC inhibition to promote neuronal autophagy and clearance of misfolded proteins (42, 151).

The relationship between gut microbiota and PD pathology is complex. Alpha-synuclein (α-syn) aggregates have been identified in duodenal biopsies from patients with early and advanced PD (152), and animal models show sequential appearance of α-syn pathology in the dorsal motor nucleus of the vagus and brainstem after intramural α-syn preformed-fibril injection (153). The regulatory role of SCFAs on α-syn pathology is context-dependent and bidirectional. In germ-free α-syn-overexpressing mice, microbial colonization or SCFA supplementation worsened motor deficits and neuroinflammation (156), indicating that SCFAs should not be generalized as uniformly protective in all neurodegenerative contexts.

Probiotic supplementation and fecal microbiota transplantation (FMT) have yielded preliminary positive signals in neurodegenerative disease models: reducing systemic LPS burden, restoring SCFA production, and improving barrier function collectively attenuate neuroinflammation and improve cognitive outcomes (42, 43). Clinical translation, however, remains constrained by product heterogeneity, small trial sizes, and - for FMT - documented risk of pathogen transmission (44). Microbiota-targeted approaches should therefore be considered a promising but still investigational avenue for bone–brain co-management.

### 6.4 Metabolic syndrome and the synergistic effects of insulin resistance

Metabolic syndrome (MetS) - central obesity, hypertriglyceridaemia, reduced HDL, hypertension, and impaired fasting glucose - has insulin resistance (IR) as its core pathogenic driver. Two IR-related molecular nodes are directly relevant to bone–brain comorbidity: (i) AGEs accumulation and RAGE signaling in bone; and (ii) constitutive GSK-3-beta activation and impaired IDE-mediated Abeta clearance in the brain. Other MetS-related pathologies (dyslipidaemia, hypertension) act indirectly through shared vascular and inflammatory pathways described elsewhere in this review (154).

In bone, advanced glycation end products (AGEs) accumulate in collagen under hyperglycaemia, disrupting cross-linking and material mechanical properties; engagement of RAGE activates oxidative stress and NF-kB, suppressing osteoblast function and independently increasing fracture risk even when BMD is preserved (155). Osteoblast insulin signaling (IRS-1/PI3K/Akt) supports Runx2-dependent osteogenesis; IR impairs this axis, biases MSCs toward adipogenesis, and shifts cellular metabolism toward glycolysis, together constituting the cellular basis of diabetic osteoporosis (155, 157).

In the brain, impaired insulin signaling is a well-established mechanism in AD. Reduced PI3K/Akt activity releases GSK-3-beta from inhibitory phosphorylation, driving constitutive activation and tau hyperphosphorylation beyond the capacity of the proteasomal system (158). Hyperinsulinaemia additionally competes for insulin-degrading enzyme (IDE), reducing IDE-mediated Abeta clearance and accelerating amyloid deposition (158). Epidemiological meta-analyses report elevated all-cause dementia and AD risk in diabetes (RR ~ 1.56 - 1.73), consistent with - but not proof of - an independent causal association (159).

**Figure 5:**
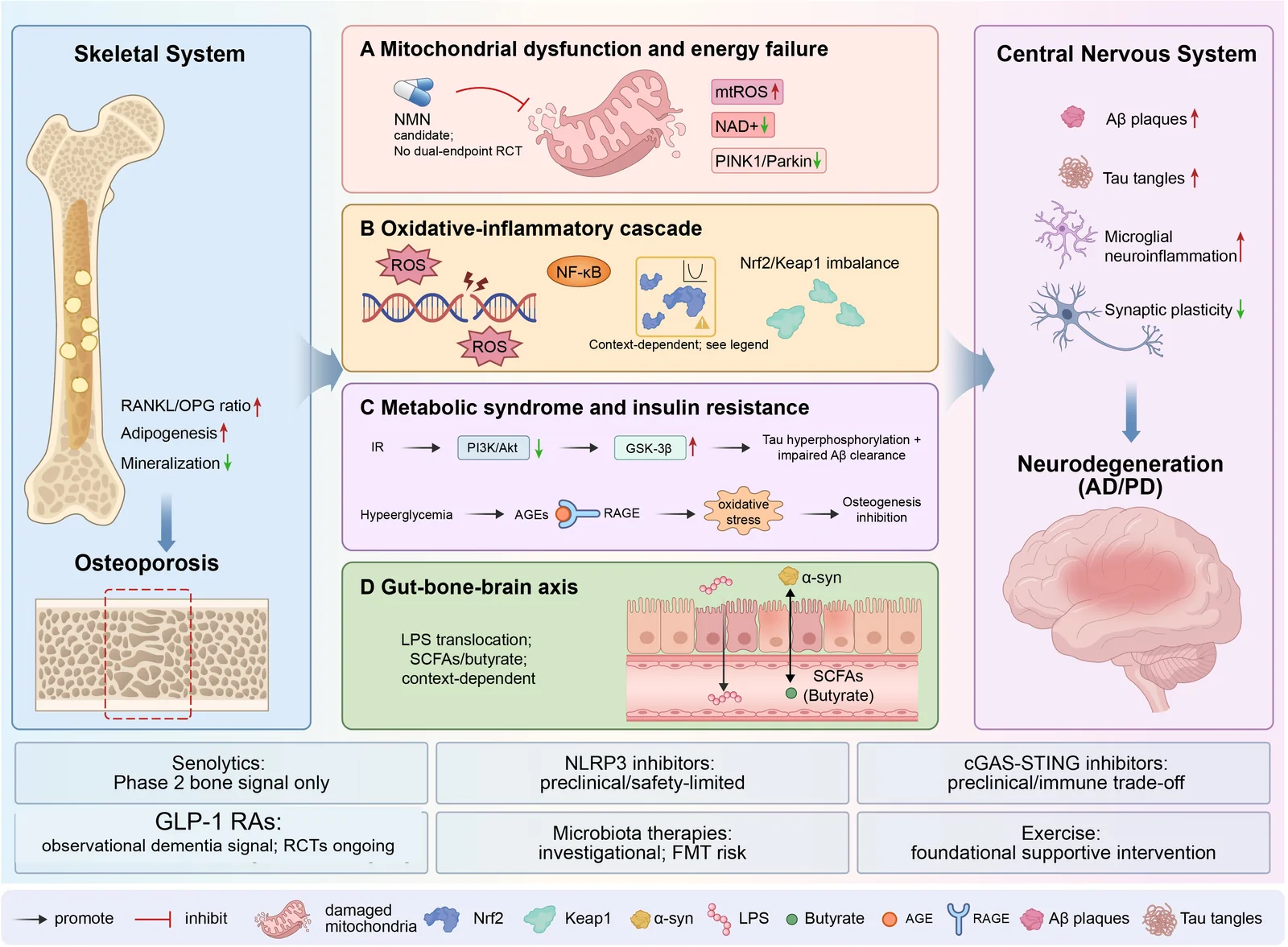
Common pathological hubs linking skeletal aging and neurodegeneration, with evidence-level and therapeutic-caution annotations. The central framework groups four cross-organ mechanisms: **(A)** mitochondrial dysfunction and energy failure, including mtROS elevation, NAD+ decline, and impaired PINK1/Parkin mitophagy; **(B)** oxidative-inflammatory cascades involving ROS, NF-κB, and context-dependent Nrf2/Keap1 imbalance; **(C)** metabolic syndrome and insulin resistance, including impaired PI3K/Akt signaling, AGE/RAGE activation, GSK-3β upregulation, impaired Aβ clearance, and osteogenesis inhibition; and **(D)** the gut–bone–brain axis, including LPS translocation, SCFA/butyrate signaling, and context-dependent microbiome effects. The lateral panels summarize skeletal outputs (RANKL/OPG ratio, adipogenesis, mineralization, osteoporosis) and CNS outputs (Aβ plaques, tau tangles, microglial neuroinflammation, synaptic plasticity, neurodegeneration). The bottom boxes emphasize that candidate interventions remain unevenly supported: senolytics have a Phase 2 bone signal only, NLRP3 and cGAS-STING inhibitors remain preclinical or safety-limited, GLP-1 receptor agonists have observational dementia signals with RCTs ongoing, microbiota-targeted therapies remain investigational, and multimodal exercise is the foundational supportive intervention.

GLP-1 receptor agonists and SGLT-2 inhibitors have been associated with reduced dementia risk in observational analyses and confer favorable bone-metabolic profiles (158, 160). Adiponectin, reduced under MetS conditions, signals through AdipoR1/AMPK to inhibit both osteoclast differentiation and M1-polarised microglial activation. These findings position IR as a convergent metabolic axis of bone–brain co-pathology, though prospective dual-endpoint trials remain necessary before clinical recommendations can be made. **Figure 5** integrates the four pivotal mechanistic hubs: mitochondrial dysfunction, oxidative stress, metabolic/insulin resistance, and the gut–bone–brain axis.

## 7 Conclusions and future directions

### 7.1 Summary of core arguments: inflammaging as the central mechanistic hub linking skeletal aging and cognitive impairment

This review has examined the comorbid relationship between osteoporosis and neurodegenerative disease, tracing convergent pathological pathways from epidemiology to molecular mechanism. Meta-analytical evidence indicates that osteoporosis is associated with a substantially elevated risk of cognitive impairment (OR = 2.01; 95% CI 1.63-2.48) (161), and that cognitive impairment carries a reciprocally increased risk of osteoporosis (RR = 1.56; 95% CI 1.30-1.87) - with AD conferring a particularly high risk (RR = 1.70; 95% CI 1.23-2.37) (6). This bidirectional association is best interpreted as the shared output of multiple interlocking aging mechanisms rather than as a direct causal relationship between the two conditions; the direction and magnitude of any residual causal link require further human-genetic and prospective validation.

Among the shared drivers identified, inflammaging - the chronic, low-grade, sterile inflammatory state sustained by senescent cells and their SASP - occupies the most central mechanistic position and is recognized as a canonical hallmark of aging (162). Through sustained secretion of IL-6, IL-1beta, and TNF-alpha, SASP simultaneously activates RANKL-dependent osteoclastogenesis and drives persistent microglial activation, exacerbating Abeta aggregation, tau hyperphosphorylation, and neuronal apoptosis (162, 163). Mitochondrial dysfunction amplifies SASP output through cytoplasmic DNA release and cGAS-STING activation, establishing a “senescence-inflammation-tissue injury” cascade (163). Beyond this inflammatory axis, bone-derived endocrine signals - most prominently OCN, SOST, and FGF23 - have been proposed to participate in bone–brain communication; as discussed in Sections 5.2.1-5.2.3, the strength of clinical evidence differs across these molecules and none currently meets the threshold of an established therapeutic axis in humans (115).

#### 7.1.1 Key take-home messages

1. Osteoporosis and cognitive decline are associated in both directions, but this does not establish direct bidirectional causation. Shared upstream drivers, including age, sarcopenia, vascular disease, endocrine status, metabolic health, and lifestyle exposures, probably explain part of the observed coupling.
2. Inflammaging provides the most parsimonious shared upstream mechanism. Chronic sterile inflammation driven by senescent cells and their SASP can activate RANKL-dependent osteoclastogenesis in bone while priming microglial neuroinflammation in the central nervous system.
3. The endocrine arm of the bone–brain axis remains biologically plausible but clinically unsettled. Evidence for OCN, SOST, and FGF23 differs in strength, and each pathway carries unresolved translational or confounding issues.
4. No pharmacological agent currently satisfies the evidentiary threshold for bone–brain co-therapy. GLP-1 receptor agonists, senolytics, NLRP3 inhibitors, cGAS-STING inhibitors, and microbiota-targeted approaches all require more direct dual-endpoint evidence.
5. The integrated bone–brain management framework proposed here is aspirational. It should be treated as a testable model for risk stratification and trial design, not as an evidence-based standard of care.

### 7.2 Current limitations, unresolved questions, and translational challenges

In place of the brief limitations paragraph in the original manuscript, this section provides a substantive assessment organized into (7.2.1) methodological limitations, (7.2.2) unresolved mechanistic questions, (7.2.3) translational barriers, (7.2.4) selected controversies, and (7.2.5) shared upstream drivers and confounding.

#### 7.2.1 Methodological limitations of the current evidence base

The epidemiological literature linking osteoporosis and cognitive decline is dominated by cross-sectional or short-follow-up cohort designs, which cannot distinguish shared upstream aging from directional causation. Heterogeneity in outcome definitions - BMD versus clinical osteoporosis, MCI versus all-cause dementia versus AD - and inconsistent adjustment for shared risk factors (physical activity, vitamin D, sex-hormone status) further limit inference. Adequately powered Mendelian-randomisation studies with strong genetic instruments for BMD, OCN, SOST, or FGF23 as exposures and cognitive endpoints as outcomes remain scarce (6, 161).

At the mechanistic level, most of the evidence supporting the inflammaging-bone–brain framework is derived from rodent models with a compressed aging trajectory; human validation is largely restricted to biomarker association studies and to a small number of early-phase interventional trials. The evidentiary basis for the framework should therefore be characterized as strong for preclinical mechanism and limited for direct human causation.

#### 7.2.2 Unresolved mechanistic questions

Four questions require particular attention. **First**, the quantitative contribution of bone-derived endocrine signals (OCN, SOST, FGF23) to human cognitive function, relative to other systemic mediators, has not been partitioned. **Second**, whether the cGAS-STING axis operates as a *driver* of age-related neuroinflammation in humans, or as a *downstream marker* of mitochondrial dysfunction, is unresolved (22). **Third**, the causal role of specific microglial states - DAM subclusters, IFN-responsive states, lipid-associated microglia - in progression from inflammaging to cognitive decline remains descriptive rather than causally established (94, 95). **Fourth**, the temporal ordering of osteocyte senescence, SASP emergence, and systemic inflammaging in humans is largely inferred from cross-sectional biomarker studies rather than from longitudinal tissue-level measurements (164).

#### 7.2.3 Translational barriers

Translation from mouse to human is impeded by (i) well-recognized species differences in the immunosenescence trajectory; (ii) the difficulty of achieving CNS exposure with peripherally administered anti-inflammatory agents - BBB penetration is limited for MCC950, the dasatinib+quercetin (D+Q) senolytic combination, and most cGAS-STING candidates; (iii) the absence of validated intermediate biomarkers that would allow proof-of-concept trials shorter than a fracture- or dementia-endpoint trial; and (iv) the need for **dual primary endpoints** (bone and cognitive) in trial design, which few sponsors currently pursue. Long-term senolytic administration in older adults with limited safety data raises additional ethical constraints on trial design.

#### 7.2.4 Selected controversies

These include: the *Bglap*/*Bglap2* osteocalcin knockout controversy discussed in Section 5.2.1 (17, 18); the bidirectional role of Nrf2 in bone in Section 6.2 (165); the context-dependent effects of SCFAs on alpha-synuclein aggregation in Section 6.3 (156); the discordance between observational associations of GLP-1 receptor agonists with reduced dementia risk and the awaited randomized evidence (40); and the debate over whether adult hippocampal neurogenesis persists at a magnitude sufficient to be modulated therapeutically in humans (112).

#### 7.2.5 Shared upstream drivers and confounding: implications for causal interpretation

A recurrent difficulty in interpreting the epidemiological bone–brain association is that skeletal and cognitive aging share a large set of upstream drivers, many of which are inconsistently adjusted for in the primary literature. These include chronological age, physical inactivity, sarcopenia, vascular disease, endocrine status (sex-hormone loss and thyroid dysfunction), metabolic health (type 2 diabetes, insulin resistance, and adiposity), vitamin D and vitamin K status, glucocorticoid exposure, anti-epileptic and psychotropic medication use, sensory impairment contributing to both fall risk and dementia risk, and socioeconomic factors including nutrition and educational attainment. In many cohorts, these covariates are adjusted for individually but not as a shared latent structure, and residual confounding can produce an apparent bone-cognition association even without a direct causal link. Mendelian randomization does not yet fully resolve this problem because available genetic instruments for BMD may have pleiotropic links to vitamin D metabolism, growth-axis biology, or body composition. The causal architecture of the bone–brain association should therefore be read at present as shared upstream drivers with a possible additional direct pathway, rather than as proof of direct bidirectional causation. The framework advanced in this review does not require the direct pathway to be dominant; it requires only that inflammaging and its downstream effectors constitute a shared, testable upstream node.

### 7.3 Future research directions and critical evaluation of candidate therapies

The previous version of this subsection presented several dual-action interventions with insufficient critical assessment. In response to reviewer feedback, we now provide (i) a research-directions summary followed by (ii) a structured evaluation of candidate therapies with explicit evidence-level and safety framing.

#### 7.3.1 Research priorities

Multi-omics integration offers a pathway to identify cross-tissue regulatory networks and robust biomarker signatures for bone–brain comorbidity. Single-cell sequencing can reveal inflammaging-driven transcriptional-state transitions across osteoblasts, osteoclasts, and microglia; circulating proteome studies offer a complementary approach to identifying shared systemic protein signals (163).

Large-scale longitudinal cohort studies are indispensable for establishing temporal causal architecture. Future cohorts should incorporate synchronized serial measurements of BMD, bone-turnover markers (P1NP, beta-CTX), standardized cognitive assessment (MoCA, MMSE), inflammatory biomarkers (IL-6, CRP), and bone-derived hormones (OCN, SOST), together with biobanking to support mechanistic substudies (6, 161).

#### 7.3.2 Critical evaluation of candidate bone–brain dual-targeting interventions

The interventions summarized in **Table 1** share a conceptual attractiveness: by targeting a shared upstream inflammatory driver, they might in principle benefit both skeletal and cognitive endpoints. However, none currently satisfies the evidentiary threshold required for clinical recommendation in the bone–brain comorbidity context. Direct human data are strongest for GLP-1 receptor agonists (although the strongest signals are observational and dementia RCTs are ongoing) and for the D+Q senolytic combination on bone endpoints (a single subgroup-driven Phase 2 signal). All remaining candidates rest on preclinical foundations with unresolved safety questions - hepatotoxicity for first-generation NLRP3 inhibitors, immune-competence trade-offs for chronic cGAS-STING blockade, and product heterogeneity for microbiota-based approaches. Dual-endpoint randomized trials, powered separately for bone and cognitive outcomes and enriched for individuals with elevated inflammaging biomarkers, are needed before any of these agents can be positioned as bone–brain co-therapies.

Non-pharmacological approaches retain a shared physiological rationale for both organ systems. A large meta-analysis (104 studies, > 341,000 participants) reported a positive association between physical activity and global cognitive function in older adults, with domain-specific benefits for episodic memory and verbal fluency (45). Multimodal exercise programs remain the most evidence-supported foundational component of any integrated bone–brain management approach (166).

### 7.4 Clinical implications: advancing an integrated bone–brain management paradigm

The evidence synthesized in this review carries potential implications for clinical research, risk stratification, and interdisciplinary assessment. Under traditional disease-management models, osteoporosis and neurodegenerative disorders are managed in organizational isolation across distinct specialties. However, the convergence of the two conditions at the levels of molecular mechanism and shared risk factors provides a rationale for testing integrated bone–brain management models rather than for immediate practice-changing recommendations (19, 167).

For the purposes of this review, we define bone–brain integrated management as a coordinated clinical framework in which older adults undergo (i) synchronous baseline screening for skeletal (DXA-derived BMD, bone-turnover markers) and cognitive (MoCA/MMSE) status; (ii) cross-referral pathways ensuring that individuals diagnosed with one condition receive assessment for the other; and (iii) shared decision-making regarding therapies whose benefit-risk profile spans both domains (e.g. GLP-1 receptor agonists, multimodal exercise). We emphasize that this framework is currently aspirational: no professional-society guideline yet endorses it, no reimbursement pathway supports it, and its outcome benefit has not been established in randomized trials. It is proposed here as a hypothesis-generating clinical model, not as an evidence-based standard of care.

At the level of clinical screening, concurrent assessment of bone-metabolic status and cognitive function is a reasonable candidate for adults aged 60 years and older, given that cognitive impairment amplifies fall and fragility-fracture risk while post-fracture immobilization and analgesic requirements can accelerate cognitive deterioration (6, 167). Patients with established bone–brain comorbidity warrant high-risk surveillance pathways attentive to the interactive dynamics of fall-fracture risk and cognitive trajectory.

At the level of therapeutic decision-making, no single agent currently meets the evidentiary standard for dual bone–brain indication (see **Table 1**). Where a therapeutic decision must be made - for example, glycaemic therapy selection in an older adult with osteoporosis, or antiresorptive selection in a patient with early cognitive impairment - the framework outlined above supports shared decision-making that explicitly considers effects on both organ systems, rather than optimizing each condition in isolation. Multimodal exercise remains the intervention with the strongest cross-organ evidence and should be the foundational non-pharmacological component of integrated management (45).

In conclusion, the comorbidity of osteoporosis and neurodegenerative disease is not a clinical coincidence but a plausible consequence of systemic aging-associated biological damage, in which inflammaging appears to play a central integrative role. The framework advanced in this review is intended to *consolidate a fragmented literature into an integrative pathophysiological perspective* and to define a research and clinical agenda, rather than to establish a new evidence-based standard of care. Realizing this agenda will require cross-specialty collaboration among orthopedics, neurology, geriatrics, and endocrinology, together with dedicated dual-endpoint trials designed to test the framework itself.
